# Unveiling iodine-based electrolytes chemistry in aqueous dye-sensitized solar cells†
†Electronic supplementary information (ESI) available: Speciation data, spectral analysis of aqueous electrolytes, fitted EIS parameters, *J*–*V* and stability curves of assembled DSSCs, MATLAB^®^ code to reproduce our data analysis. See DOI: 10.1039/c6sc01145d


**DOI:** 10.1039/c6sc01145d

**Published:** 2016-04-13

**Authors:** F. Bella, S. Galliano, M. Falco, G. Viscardi, C. Barolo, M. Grätzel, C. Gerbaldi

**Affiliations:** a GAME Lab , CHENERGY Group , Department of Applied Science and Technology – DISAT , Politecnico di Torino , Corso Duca degli Abruzzi 24 , 10129-Torino , Italy . Email: federico.bella@polito.it ; Email: claudio.gerbaldi@polito.it; b Department of Chemistry and NIS Interdepartmental Centre , Università degli Studi di Torino , Via Pietro Giuria 7 , 10125-Torino , Italy . Email: claudia.barolo@unito.it; c Laboratory of Photonics and Interfaces , Institut des Sciences et Ingénierie Chimiques , Ecole Polytechnique Fédérale de Lausanne (EPFL) , Station 3 , CH1015-Lausanne , Switzerland

## Abstract

Aqueous dye-sensitized solar cells (DSSCs) have recently emerged as promising systems, which can combine low cost and environmental compatibility with appreciable efficiency, long-term durability and enhanced safety. In the present study, we thoroughly investigate the chemistry behind the iodide/triiodide-based redox mediator, which presents – in a completely aqueous environment – several differences when compared to the behavior observed in the conventionally used organic solvents. The speciation of ions, the effect of the concentration of the redox mediator and the type of counter-ion are characterized from the electrochemical, spectroscopic, photovoltaic and analytical viewpoints. Furthermore, we demonstrate that aqueous DSSCs, often assumed as unstable, hold the potential to assure unparalleled stability after five months of aging without any addition of stabilizers or gelling agents, thus envisaging the construction of eco-friendly photovoltaic devices free of expensive, flammable and toxic solvents.

## Abstract 2

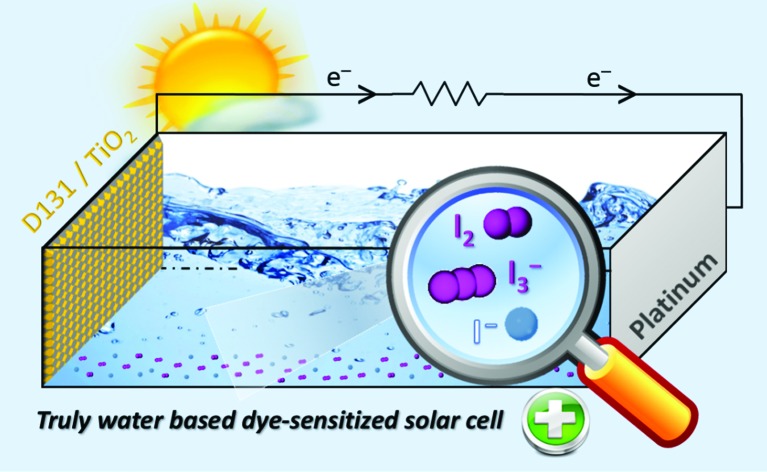
The chemistry behind the I^–^/I_3_^–^ redox couple is thoroughly investigated in 100% aqueous dye-sensitized solar cells, paving the way to this emerging green PV technology.

## 1. Introduction

At the same time that perovskite solar cells are continuously breaking high efficiency records in the recent literature reports, a diverse third-generation technology is establishing itself in the photovoltaic research community: aqueous solar cells.^1^ These devices are conceived to overcome one of the main limitations of standard dye-sensitized solar cells (DSSCs),^2^–^4^ *viz.* the use of high vapour pressure, toxic and flammable organic solvents-based electrolytes. Even if solid-state hole-transporting materials were proposed in perovskites and DSSCs to replace liquid electrolytes, their cost remains too high for a widespread diffusion of this technology (*e.g.*, spiro-OMeTAD costs 450 k$ kg^–1^).^5^ On the other hand, introducing water in DSSCs would assure the realization of cheap, non-flammable (thus safe) and truly eco-friendly solar conversion devices, at the same time overcoming their well-known poor stability upon moisture/water contamination. Indeed, several literature studies demonstrated that water is a poisoner for standard DSSCs: it causes the negative shift of the TiO_2_ conduction band,^6^,^7^ the weakening of the TiO_2_–dye interaction (eventually, up to dye desorption),^8^,^9^ the photoinduced substitution of the –NCS ligand (and related changes in the absorption properties of the sensitizer),^10^,^11^ the disappearance of I_3_^–^ ions in the electrolyte,^12^ and the unwelcome interaction with both additives and salts in the liquid redox mediator solution.^13^ All of these phenomena are even more aggravated when large-scale application of DSSCs is conceived.

Aqueous DSSCs have recently scored an interesting 5.64% efficiency,^14^ and the present research efforts are addressed towards the improvement of the wettability of the photoanode,^14^,^15^ the formulation of new water-soluble redox mediators,^16^–^19^ the synthesis of water-resistant organic sensitizers,^20^–^22^ and the preparation of new counter-electrodes.^23^ Nevertheless, many articles on this emerging topic attempt to export the expertise gained on traditional DSSCs to aqueous devices, which often results in lack of reliability. Actually, several literature reports show aqueous DSSCs based on the ruthenium N719 dye,^24^,^25^ although it was already verified that its –COOH moieties connecting to the TiO_2_ electrode are hydrolyzed by water in a few seconds, leading to cell failure in less than a minute.^1^ In the same way, redox mediator salts are often used in aqueous electrolytes in the same concentrations adopted in the presence of organic solvents,^24^,^26^ although the solubility in water is surely higher; similarly, some organic/apolar additives typical of traditional electrolytic systems are mixed with water^20^,^27^ even if after a few minutes a phase separation likely occurs. Furthermore, reading the literature it is easy to get confused between aqueous DSSCs and water-based DSSCs, the latter being cells based on electrolytes that contain a 10–60 vol% of water in the redox mediator solution,^28^–^30^ thus resulting in hybrid systems which are difficult to be contextualized with respect to state of the art. Overall, we think that the most reasonable pathway would be that of moving towards 100% aqueous electrolytes, so completely avoiding the presence of organic solvents in solar cells.

In this work, we focus on the iodide/triiodide system that is the redox mediator of choice for aqueous DSSCs when cheap and heavy metals-free technologies are envisaged. We tailored a speciation model useful to identify all of the species present in the iodine-based truly aqueous electrolyte. The effect of the concentration of the redox mediator (both in its oxidized and reduced components) as well as the effect of two different counter-ions are investigated in a wide experimental domain. After identifying their optimal operating conditions, the aqueous DSSCs based on this redox couple are subjected to long-term aging tests (exceeding five months). No additives are used in all the stages of the investigation, which allows us to comprehensively unravel the iodine-based redox mediator characteristics in terms of photovoltaic parameters, degradative processes upon time and electrochemical parameters measured by impedance spectroscopy. Based on the study detailed in the present work, a convincing prospectus of iodine-based truly aqueous electrolytes clearly comes into sight.

## 2. Experimental

### 2.1 Materials

Sodium iodide (NaI), potassium iodide (KI), iodine (I_2_), chenodeoxycholic acid (CDCA), ethanol (EtOH), acetone, *tert*-butanol (*t*-BuOH) and acetonitrile (ACN) were purchased from Sigma-Aldrich. Deionized water (DI-H_2_O, 18 MΩ cm^–1^ at 25 °C) was obtained with a Direct-Q 3 UV Water Purification System (Millipore).

Sensitizing dye 2-[{4-[4-(2,2-diphenylethenyl)phenyl]-1,2,3,3*a*,4,8*b*-hexahydrocyclopento[*b*]indole-7-yl}methylidene]cyanoacetic acid (D131) was purchased from Inabata Europe S.A.

FTO-glass plates (sheet resistance 7 Ω sq^–1^, purchased from Solaronix) were cut into 2 cm × 1.5 cm sheets and used as substrates for the fabrication of both photoanodes and counter-electrodes.

For comparison purposes, EL-HSE (High Stability Electrolyte, Dyesol) was used as a representative organic solvent-based liquid electrolyte.

### 2.2 Preparation and speciation of electrolytes

Liquid electrolytes at fixed 50 mM I_2_ were prepared by dissolving I_2_ and different amounts of KI or NaI in DI-H_2_O. The following concentrations of iodide (both K^+^ and Na^+^) salts were selected for liquid electrolytes preparation: 0.50, 1.50, 2.50, 3.50, 4.50, 5.50 and 6.50 M, except KI 6.50 M because of salt precipitation. Stock solutions were diluted when lower I_2_ concentrations are needed. In this respect, electrolytes containing I_2_ 25 mM and I^–^ 2.75 M are obtained diluting 1 : 2 (v/v) electrolytes containing I_2_ 50 mM and I^–^ 5.50 M. Electrolytes containing I_2_ 25 mM and I^–^ 5.50 M were freshly prepared, instead. All of the electrolyte solutions were stored in glass bottles under dark condition.

Stock solutions were diluted to the required concentration before recording each UV-vis spectrum at different times. 1 : 2000 (v/v) dilution allowed displaying signals due to I^–^ as well as I_3_^–^ in the case of solutions containing I^–^ 0.50 M and I_2_ 50 mM. In all other cases, further dilution did not allow both signals to be displayed accurately. Consequently, we focused only on the I_3_^–^ signals to monitor the electrolyte solution, despite the saturation of the I^–^ peak. The spectra of all the electrolyte solutions in dilution 1 : 1000 (v/v) were also recorded after 12 and 22 days (KI-containing solutions) or 13 and 22 days (NaI-containing solutions).

UV-vis absorption spectra were recorded using a CaryBio 300 (Varian) spectrometer collecting one point every 0.5 nm in the double range mode using quartz cuvettes. The error was estimated by computing the standard deviation related to the absorption maxima of six distinct samples prepared diluting at 1 : 1000 (v/v) the electrolyte solution containing I^–^ 0.50 M and I_2_ 25 mM.

**Fig. 1:**
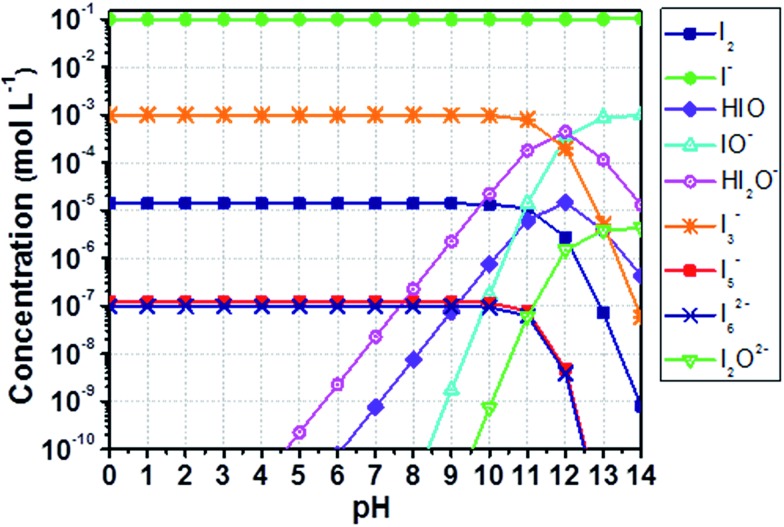
Computed concentration of iodine compounds in a solution containing aqueous I^–^ 0.10 M and I_2_ 1.0 mM as a function of the pH. Results are computed as described in this section, comparable with those obtained by Gottardi (see Fig. S1†).^31^ Lines just connect data points for better reading and have no physical meaning.

The equilibrium concentrations of I_2_, I^–^, I_3_^–^, higher polyiodides and species following from the formation of hypoiodous acid (HIO) were computed by means of a thorough speciation analysis. The reader is invited to go through Appendix 1† for a complete comprehension of the proposed scheme. The method accuracy was evaluated comparing computed data (Fig. 1) with those obtained by Gottardi (see Fig. S1†).^31^ MATLAB^®^ software was used for computations. Instructions are detailed in Appendix 2.†

### 2.3 Fabrication and characterization of aqueous DSSCs

FTO covered glasses were rinsed with acetone and ethanol in an ultrasonic bath for 10 min. Then, solvent traces were removed by flash evaporation on a hotplate at 450 °C. Front electrodes were prepared by depositing a single layer of porous TiO_2_ on top of conductive substrates by means of a manual screen printer equipment with a 43T mesh frame. After deposition of the paste (18NR-T, Dyesol) and 20 min standing to let it thoroughly bed, the TiO_2_ layer was dried at 80 °C for 20 min; it was finally sintered increasing the temperature up to 480 °C in 45 min. The fabricated photoanodes displayed a thickness of ≈6 μm and a covered area of 0.25 cm^2^. They were finally reactivated by heating at 450 °C for 20 min and, subsequently, soaked at 70 °C into a D131 dye solution (0.50 M in *t*-BuOH : ACN 1 : 1). CDCA 0.90 mM was added in the dye solution as coadsorbent.^32^ Dipping in dye solutions was carried out at 22 °C for 5 h under dark conditions and shaking in a Buchi Syncore platform equipped with a cooling plate. After dye loading, photoanodes were washed in acetone to remove residual dye not specifically adsorbed on the TiO_2_ layer. As regards counter-electrodes preparation, FTO conductive glasses were platinized by spreading a H_2_PtCl_6_ solution on the plate surface and heating up to 400 °C.

Photoanodes were faced to the counter-electrodes exploiting Surlyn thermoplastic frames (internal area 0.6 cm × 0.6 cm) as spacers (60 μm thick), taking care of the overlapping of the active areas. All of these components were assembled by hot pressing at 110 °C for 20 s. The electrolyte solution was injected by a vacuum backfilling process through a hole in the counter electrode, which was then sealed by a commercial epoxy glue.^33^

Photovoltaic performances were evaluated recording three consecutive *J*–*V* curves on a Keithley 2420 Source Measure Unit. Cells were irradiated under simulated 1 sun light intensity (100 mW cm^–2^, AM 1.5G) after calibration by silicon diode.

Electrochemical impedance spectroscopy (EIS) data were recorded using a potentiostat (CH Instruments Inc., Model 680) in the frequency range between 10 kHz and 0.1 Hz. The amplitude of the AC signal was 10 mV. Spectra were recorded under dark conditions at applied DC potentials equal to the previously measured *V*_oc_ values under 1 sun.

In order to comprehensively investigate the characteristics of both the electrolyte and the cathode/electrolyte interface, symmetrical (dummy) cells were assembled. The impedance spectrum of a dummy cell filled with aqueous NaI 1.50 M and I_2_ 50 mM was recorded under the same EIS conditions reported above, but setting the DC potential to zero. The dummy cell was assembled simply contacting two platinized FTO coated glass electrodes separated by a Surlyn thermoplastic mask, as adopted for standard devices.

**Fig. 2:**
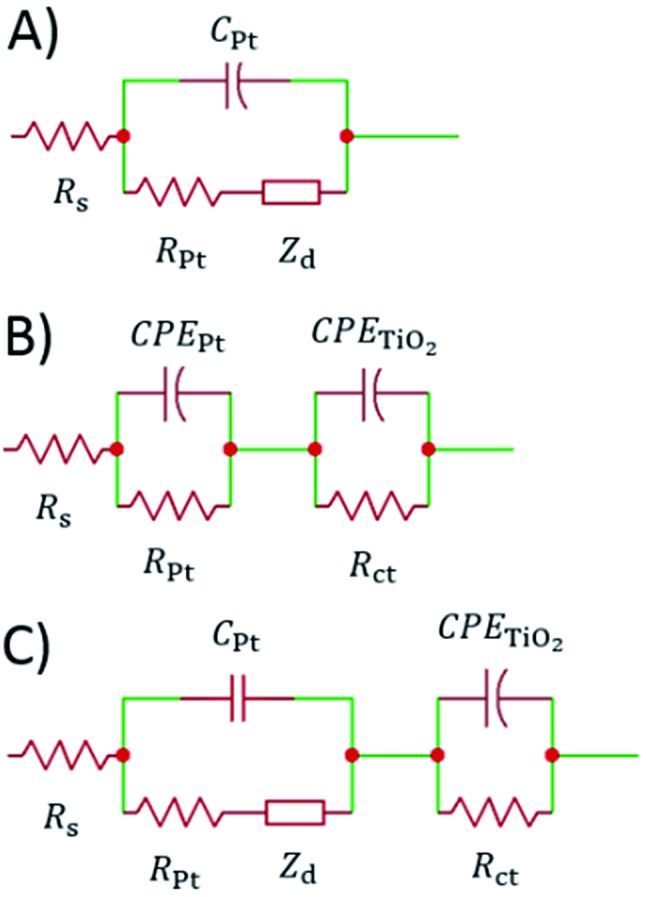
Equivalent circuits proposed for the EIS investigation of aqueous devices under study.

The impedance data were fitted using the equivalent circuits shown in Fig. 2. Their total complex impedances *Z*(*ω*) are given by the following equations:1
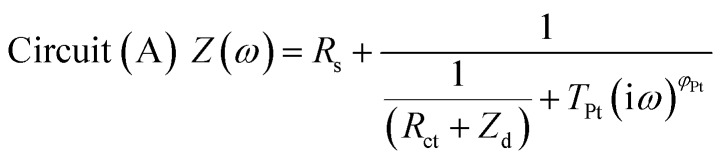

2
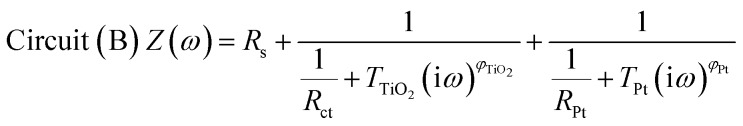

3
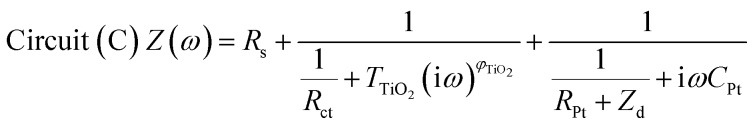
where *R*_s_ is the series resistance, *R*_ct_ is the charge transfer resistance at the TiO_2_/electrolyte interface, *Z*_d_ is the impedance due to ionic diffusion, *ω* is the frequency of the applied small-amplitude modulated potential, *T*_Pt_ and *φ*_Pt_ are the parameters of the constant phase element (CPE) used to describe the double-layer capacitance at the Pt/electrolyte interface (accounting for the corresponding depressed semicircle in the Nyquist plot), *T*_TiO_2__ and *φ*_TiO_2__ are the parameters of the CPE used to describe the double layer capacitance at the TiO_2_/electrolyte interface (accounting for the corresponding depressed semicircle in the Nyquist plot), and *C*_Pt_ is the capacitance at the Pt/electrolyte interface. Data were computed minimizing the sum of the weighted squared residual moduli (WSS):4
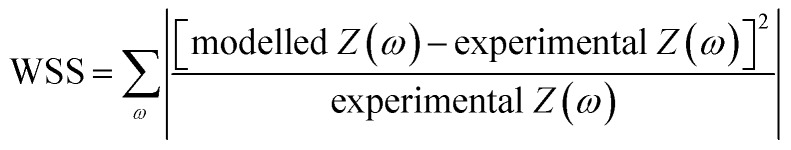



When cells were assembled with EL-HSE, signals ascribed to the electrolyte mass transport were partly embedded in the signals referred to the processes at the TiO_2_/electrolyte interface. Consequently, data were just fitted up to 1 Hz, excluding the effect of I_3_^–^ diffusion. In the case of aqueous DSSCs, the signals due to the processes at the Pt/electrolyte interface overlapped the signals due to processes at the TiO_2_/electrolyte interface. For this reason, an attempt was made to fit the data with constraints on the parameters related to the Pt/electrolyte interface or on the constant phase element *T*_TiO_2__. The constraint 0.90 ≤ *φ* ≤ 1, where *φ* stands for the CPE parameter, was applied in all cases. The double layer capacitance was calculated from the CPE as follows:^34^5
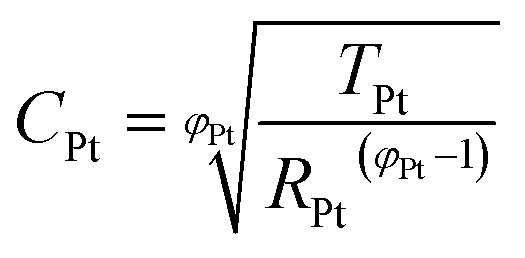

6
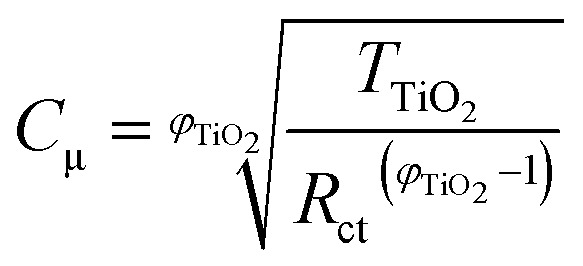



The computations were done using MATLAB^®^. Instructions are listed in Appendices 3–5.†

## 3. Results and discussion

### 3.1 Effect of iodide type and concentration on photovoltaic parameters

Initially, we investigated experimentally the effects of I^–^ concentration on the photovoltaic parameters of aqueous DSSCs sensitized with D131 dye. Since the aim of this work was to study the chemistry of the iodine-based redox mediator in aqueous solar cells, we deliberately chose not to introduce any additive (of those typically used to boost DSSCs performance) in the electrolytes. In the first part of this study, the concentration of I_2_ was kept at 50 mM in order to reasonably avoid the photocurrent to be limited by diffusion. Under these conditions, dark current generation is expected to be higher when compared to aqueous electrolytes containing only I_2_ 20 mM, which might negatively affect *V*_oc_ values. Moreover, *V*_oc_ values can further decrease upon I^–^ concentration increase, because of the Nernstian shift of the electrolyte redox potential. On the other hand, the edge position of the conduction band should not be influenced by the iodine salt concentration in aqueous media and it should be the same for all the cells when keeping the same sensitization conditions. Concentration dependent shifts would be expected in the case of specific ion adsorptions related to specific surface properties of the semiconductor and/or interactions between dye molecules and ions in the solution, which may affect the surface dipole moment.^35^,^36^

**Fig. 3:**
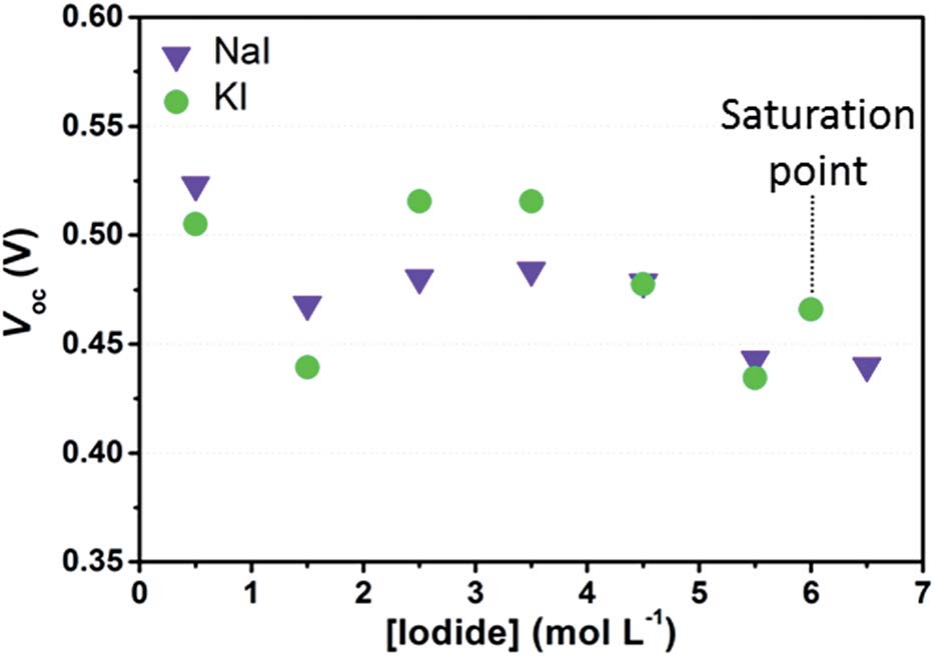
Average *V*_oc_ values recorded one day after cell assembly. I_2_ was kept constant at 50 mM for all the cells. Data are referred to the 3^rd^ measurement under 1 sun irradiation. Data referred to the electrolytes saturated with KI were arbitrarily placed at 6 M.

Fig. 3 shows the *V*_oc_ values obtained one day after assembly of cells containing I_2_ 50 mM and different I^–^ concentrations. While comparing data shown in the figure with the redox potentials of the electrolyte solutions in Table 1, one may figure out that *V*_oc_ values do not match with the expected 100 mV decrease due to the Nernstian shift of the electrolyte potentials upon I^–^ concentration increase. Moreover, considering data dispersion, no straightforward difference was evinced between NaI- and KI-based devices. As already stated, the *V*_oc_ value also depends upon the dark current. According to Law *et al.*,^15^ the rate of back electron transfer should be reduced as an effect of the decrease of the equilibrium concentration of iodine ([I_2_]_eq_, *i.e.* that is not complexed in polyiodides) while increasing the concentration of I^–^. The outcome of the experimental data enlightens that, at high I^–^ concentration, the reduction of *V*_oc_ due to the decrease of the electrolyte redox potential may have been compensated by the reduction in recombination losses. In the case of cells sensitized with D131 dye, such effect might be even larger, because this dye is able to form complexes with I_2_ in its ground state.^37^

**Table 1:** *E*(I_3_^–^/I^–^) of the electrolyte solutions

[NaI]_0_ or [KI]_0_ (mol L^–1^)	[I_2_]_0_ (mmol L^–1^)	[I_2_]_eq_^*a*^ (mmol L^–1^)	*E* (V *vs.* NHE)
0.50	50	0.15	0.53
1.50	50	0.05	0.48
2.50	50	0.03	0.46
3.50	50	0.02	0.45
4.50	50	0.02	0.44
5.50	50	0.01	0.43
6.50	50	0.01	0.43
2.75	25	0.01	0.45
5.50	25	0.01	0.42

^*a*^[I_2_]_eq_ is referred to the equilibrium concentration of iodine calculated considering I_2_ + I^–^ ↔ I_3_^–^ and assuming *K* = 723.^31^

As stated in Section 2, we also considered aqueous electrolytes bearing lower I_2_ concentration. Observing the data listed in Table 2 it can be noted that *V*_oc_ values of the cells filled with KI 2.75 M + I_2_ 25 mM were higher than all those obtained from the whole KI batch in Fig. 3. The same was observed for aqueous cells filled with NaI 2.75 M + I_2_ 25 mM if compared to NaI-based cells in Fig. 3, except in one case. Moreover, comparing data reported in Table 2, it can be observed that at I^–^ 5.50 M, *V*_oc_ values increased as a consequence of the reduction of the amount of I_2_ used in the electrolyte. The improvement of *V*_oc_ at I_2_ 25 mM, when compared to data at 50 mM, may be attributed to the decrease of the dark current. This is ascribed to the lower concentration of electron acceptors for back-electron transfer from the semiconductor to the electrolyte. Besides this, data in Table 1 show that the computed [I_2_]_eq_ was nearly the same in the electrolyte prepared with I^–^ 5.50 M + I_2_ 50 mM and in both the electrolytes with I_2_ 25 mM. These data more likely suggested that under these experimental conditions, *V*_oc_ can be improved by decreasing the overall concentration of I_2_, regardless of [I_2_]_eq_.

**Table 2:** Best performances of aqueous DSSCs bearing different I^–^ and I_2_ concentrations. Cells were tested under 1 sun irradiation and the reported data were recorded two days after assembly and at the 3^rd^*J*–*V* measurement

[I^–^] (mol L^–1^)	[I_2_] (mmol L^–1^)	*V*_oc_ (V)		*J*_sc_ (mA cm^–2^)		FF		PCE (%)	
		NaI	KI	NaI	KI	NaI	KI	NaI	KI
2.75	25	0.506	0.593	1.21	0.89	0.50	0.55	0.30	0.29
5.5	25	0.506	0.473	0.82	0.41	0.58	0.52	0.24	0.10
5.5	50	0.438	0.427	2.34	3.01	0.58	0.57	0.59	0.73

Electrolyte	*V*_oc_ (V)	*J*_sc_ (mA cm^–2^)	FF	PCE (%)
EL-HSE	0.722	6.12	65.3	2.88
NaI 5.5 M	0.702	0.35	48.1	0.12
KI 5.5 M	0.689	0.45	54.0	0.17

Interestingly, the average values of *V*_oc_ in Fig. 3 are low when compared to the reference cells filled with an organic solvent-based electrolyte, such as EL-HSE, that may achieve a *V*_oc_ equal to 700 mV. Such a high value might be achieved also by aqueous DSSCs loaded with electrolytes containing I^–^ 5.50 M, without I_2_. In this case, dark current is expected to approach zero, but the photocurrent would be of course strongly limited by low ionic diffusion. As is clearly evident in Table 2, these cells displayed low efficiencies; however, these data were useful to confirm that in standard aqueous DSSCs (Fig. 3) the dark current is likely to be the main factor responsible for the relatively low *V*_oc_ values.

Data listed in Table 2 show that, at fixed I^–^ 5.50 M, *J*_sc_ values decreased with decreasing the amount of I_2_ used to prepare the electrolyte. We may ascribe the decrease of this parameter to the fact that if the initial concentration of I_2_ is low, the photocurrent is limited by I_3_^–^ diffusion.

**Fig. 4:**
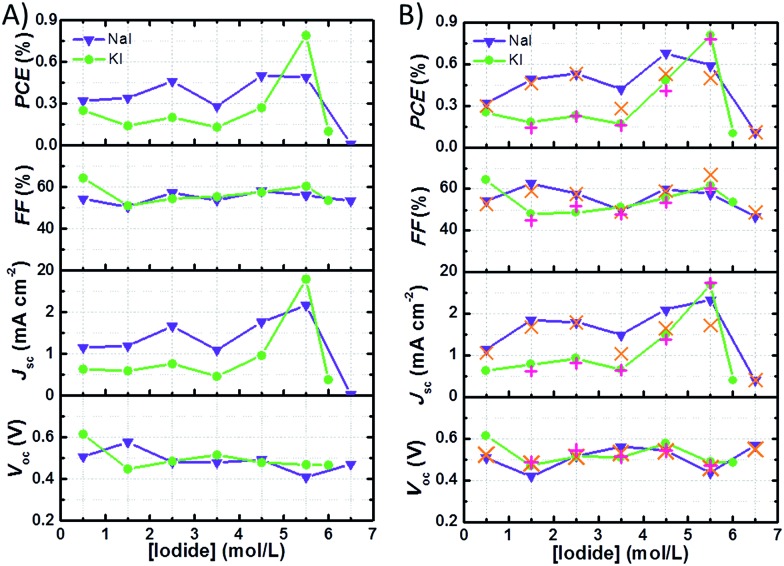
Photovoltaic parameters ((A) after 24 h from cell assembly, (B) best value recorded in the cell lifetime) of aqueous DSSCs bearing different I^–^ concentrations (I_2_ 50 mM). Orange crosses (×) or red plus signs (+) correspond to the average values at the given NaI or KI concentrations, respectively. Lines simply connect data points and have no physical meaning. Cells were tested under 1 sun irradiation and the reported data were recorded at the 3^rd^*J*–*V* measurement.

Fig. 4A shows the data referred to the best performances of the cells with I_2_ 50 mM recorded 24 h after assembly. The trend of *J*_sc_ is the main factor affecting the efficiency profile at different I^–^ concentrations. At I_2_ 50 mM, in both NaI and KI batches, the highest *J*_sc_ values were registered at I^–^ 5.50 M. Anyway, in the case of NaI-laden cells, the best performance was observed at NaI 4.50 M, which assured slightly higher *V*_oc_ and FF values. With regard to the NaI batch at I_2_ 50 mM, PCE values increased in the order of NaI 0.50 M to NaI 2.50 M. With the exception of cells with NaI 3.50 M, PCE levelled off at ≈0.45% and dropped at NaI 6.50 M. In the case of cells containing KI, PCE did not exceed 0.3% up to KI 4.50 M and peaked at KI 5.50 M reaching the maximum efficiency of ≈0.8%. Cells filled with the solution saturated with KI showed PCE values approaching zero.

It can be noted that, in the presence of I_2_ 50 mM, samples from the NaI batch displayed higher *J*_sc_ and PCE values when compared to those from the KI batch up to I^–^ 4.50 M. Moreover, KI-laden cells filled with I_2_ 25 mM displayed lower photocurrents when compared to their NaI-based counterparts. On the other hand, at I^–^ 5.50 M + I_2_ 50 mM the cells containing KI performed better that those containing NaI at the same concentration. According to literature reports, aqueous solutions containing KI are less viscous than those containing NaI.^38^ Moreover, the surface tension increment is slightly lower in the case of aqueous KI.^39^ From these literature data, it may be inferred that solutions containing KI should allow faster ion diffusion and better wetting when compared to those added with NaI. Conversely, the photovoltaic performances of the cells from NaI batch were found to overtake their KI counterparts in all cases except one (5.50 M). This likely highlights possible specific interactions between Na^+^-based aqueous solutions and the dyed electrode, thus allowing better wetting of the nanostructured photoanode. Besides this, deviations from the linearity in the plot displaying the specific electrical conductance of aqueous KI- or NaI-based electrolyte *versus* the salt concentration obtained from literature data (Fig. S2†) suggest that the extent of ion pair formation is higher in the case of NaI solutions. Most probably, this helps to explain the higher performance in cells containing KI 5.50 M if compared to those with concentrated NaI. The observed drop of *J*_sc_ and PCE values at I^–^ concentrations exceeding 5.50 M could also be due to slight sealing defects in these conditions, as will be discussed later. Anyway, data listed in Table 2 referred to the electrolyte with I_2_ 25 mM suggest that the excess of I^–^ relative to I_2_ does not improve the photocurrent generated by the cells up to a certain extent. It may be due to the high concentration of uncharged neutral ions pairs: NaI^0^ and KI^0^.^40^ At high I^–^ concentrations, quenching of the excited state of the sensitizer could also affect the photovoltaic performance (as already demonstrated for Ru-based sensitizers): indeed, this process is competitive with electron injection.

The parameters referred to the best photovoltaic performances are shown in Table 3 and plotted *versus* I^–^ concentration in Fig. 4B. In most of the cases, the best photovoltaic performances were recorded several days (>10) after cell assembly. This is typical of the aqueous solar cells.^21^ Unfortunately, the majority of the literature reports only describe photovoltaic parameters measured just after cell assembly, thus making it difficult to identify the effective capacity of each device. In the present case, *V*_oc_ values often increased over time, even when efficiency dropped. This might be due to the shift of the quasi-Fermi level (E*f), because a decrease of dark current should in principle increase all of the cell parameters, other factors being unvaried. Such a shift could be connected to surface adsorbed species; for instance, it may be due to proton desorption or to the adsorption of I^–^ on the semiconductor.^41^ Further experiments would be required to characterize the interaction between the semiconductor film and the electrolyte, but they are out of the scope of this work.

**Table 3:** Best photovoltaic parameters of aqueous DSSCs having different I^–^ concentrations (I_2_ 50 mM). Cells were tested under 1 sun irradiation and the reported data were recorded at the 3^rd^*J*–*V* measurement

[I^–^] (mol L^–1^)	Day		*J*_sc_ (mA cm^–2^)		*V*_oc_ (V)		FF		PCE (%)	
	NaI	KI	NaI	KI	NaI	KI	NaI	KI	NaI	KI
0.50	1	5	1.15	0.63	0.507	0.614	0.54	0.64	0.32	0.25
1.50	37	11	1.86	0.79	0.420	0.471	0.63	0.48	0.49	0.18
2.50	12	11	1.80	0.93	0.515	0.514	0.58	0.49	0.53	0.23
3.50	10	6	1.50	0.66	0.560	0.510	0.50	0.51	0.42	0.17
4.50	2	22	2.10	1.50	0.543	0.578	0.60	0.56	0.68	0.48
5.50	2	12	2.34	2.70	0.438	0.488	0.58	0.62	0.59	0.81
6.50	5	4	0.43	0.40	0.476	0.485	0.55	0.53	0.11	0.10

### 3.2 Long-term stability of aqueous solar cells

In this work, aqueous DSSCs were sensitized with D131 dye, since this molecule showed promising stability in aqueous environment during previous investigations.^42^ This is the first ever report on the use of this dye in aqueous DSSCs. Indeed, literature reports argue that –COOH groups in cyanoacrylic units may lead to desorption of the dye, as these groups can be turned into the corresponding aldehyde after decarboxylation, as an effect of the presence of water under UV irradiation or at high temperature (≥160 °C).^43^ However, such high temperature is not reached under standard operational condition.

**Fig. 5:**
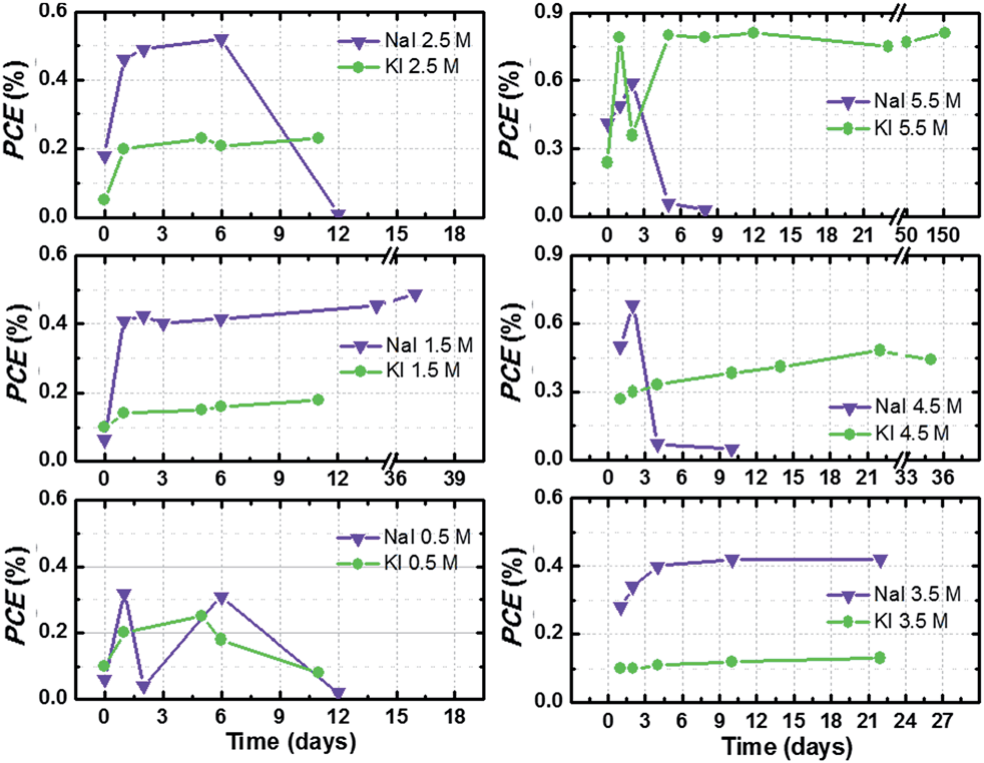
PCE values as a function of time of aqueous DSSCs assembled with different I^–^ salts and at different concentrations, keeping I_2_ constant at 50 mM. Cells (stored at room temperature under dark conditions) were tested at 1 sun irradiation and the reported data were recorded at the 3^rd^*J*–*V* measurement. Lines simply connect data points for better reading and have no physical meaning.

Efficiency values of the best performing cells are plotted *versus* time in Fig. 5. Cells were stored under dark conditions at ambient temperature and their photovoltaic performance at 1 sun was monitored over time. When efficiency values decreased below 0.3%, cell monitoring was stopped. In the other cases, efficiency was monitored for at least 20 days and little improvements over time were observed. As stated in the previous section, the behaviour upon time of aqueous DSSCs is rather different with respect to their organic solvents-based counterparts, and a sort of “activation period” is required to achieve the best efficiency value.^21^

The best performing cell filled with KI 5.50 M and I_2_ 50 mM effectively retained its 0.8% PCE for five months. Despite the fact that this performance might seem rather low when compared to some of the recent literature reports,^15^,^22^ two aspects must be underlined at this point. First, no additive was used to selectively improve PCE, because the aim of this study is to unveil the characteristics of the iodine-based redox mediator in 100% aqueous electrolytes. Moreover, almost none of the additives conventionally used in the DSSC field are soluble in water. For example, 4-*tert*-butylpyridine (TBP) and *N*-methylbenzimidazole (NMBI) are apolar compounds typically used to increase photovoltage of the cells: they are insoluble in water (also in the presence of surfactants, see Fig. S3†). Surprisingly, some research groups have recently proposed TBP- and NMBI-based aqueous DSSCs.^23^,^44^ Secondly, very few groups have published their long-term stability data so far, and typical aging times were set at 2/4/24/500 h^14^,^18^,^45^ or 2/18/20/50/75/90 days.^21^,^23^,^46^ On the contrary, we present here the longest aging tests ever conducted on an aqueous DSSC:^1^ a 156 days (3750 h) stability test to demonstrate the remarkable aging resistance achieved by iodide-based aqueous DSSCs.

Fig. S4† displays the *J*–*V* curves of the long-lasting cells recorded at different days during the aging test. *J*_sc_ slightly decreased over time with respect to its maximum value in the cell filled with KI 5.50 M + I_2_ 50 mM aqueous electrolyte; efficiency was not reduced accordingly, due to *V*_oc_ improvement. On the contrary, *J*_sc_ increased over time in the cell containing NaI 2.75 M + I_2_ 25 mM.

Overall, Fig. 5 and S4† show a particular feature of NaI-based cells: several devices abruptly stopped working, which was not the case of KI-based cells. Although the possibility of some specific effects involving sodium cannot be ruled out, sealing problems are most likely the main causes of the observed efficiency decay. As a matter of fact, we often observed (during the first or second week) that the aqueous sodium electrolyte swelled the epoxy glue used as sealant in the construction of the drilled cathodes. It was confirmed by putting a drop of uncured glue in the vicinity of a drop of NaI solution: the glue was found to turn coloured around the contact zone after several days. The reason behind this fact is currently unknown. We have recently solved this drawback by simply drying the drilled cathodes from aqueous electrolyte traces to avoid any contact between the redox mediator solution and the liquid glue. As shown in Fig. S5,† this simple trick allowed us to fabricate perfectly stable cells containing sodium-based electrolytes.

### 3.3 Photostability and spectral properties of aqueous electrolytes

In the adopted aqueous phase, the absorption spectra of the electrolytes were recorded to assess if sizeable signs of degradation could be detected (all the results are listed in Table S2†). The relatively high amount of I_2_ in aqueous electrolytes did not allow recording the spectra of the original concentrated solutions, so they had to be diluted. Dilution 1 : 500 resulted in the saturation of the I_3_^–^ signal at [I^–^] > 2.50 M, so the electrolytes were diluted 1 : 1000 before recording each spectrum. Under these conditions, signals due to I_3_^–^ could be detected at 287.5 and 351 nm, while the signal of free I_2_, which was expected at 460 nm in the vis region,^47^ could not be observed. The tail of the I_3_^–^ signal towards the vis range is likely responsible for the yellowish color of the diluted solutions. The signal of I^–^ was saturated in all cases, but further dilution was found to result in the disappearance of the I_3_^–^ signals.

**Fig. 6:**
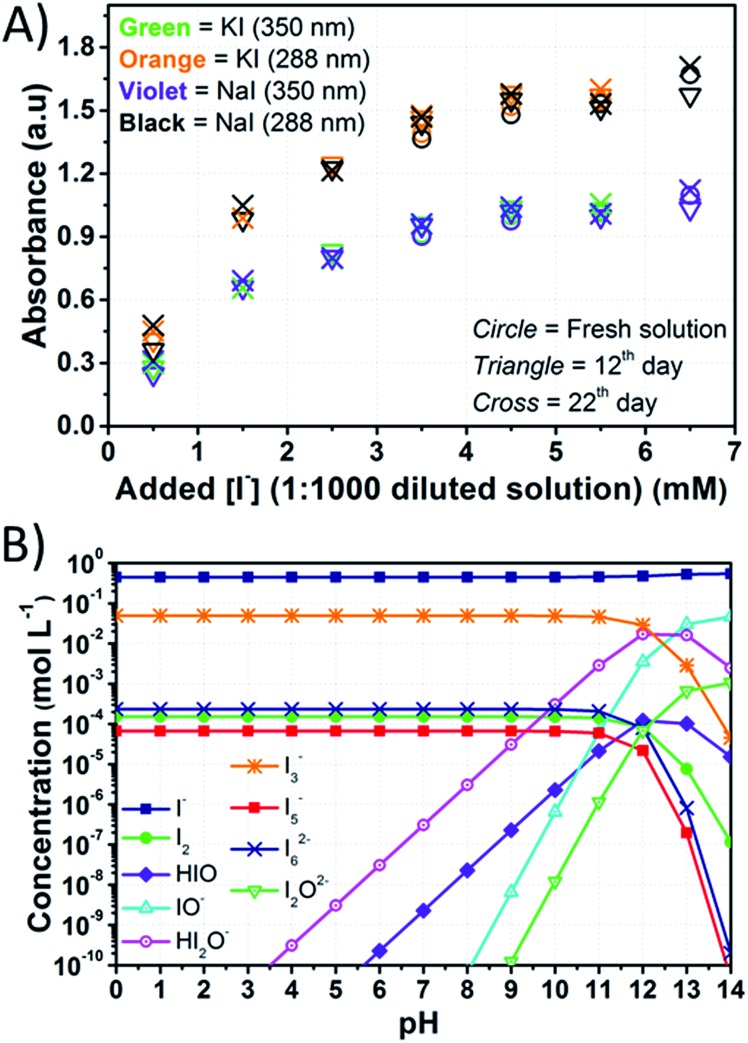
(A) Intensity of the peaks of I_3_^–^ in the UV region plotted *versus* the amount of I^–^ in the solutions obtained by diluting at 1 : 1000 the stock aqueous electrolytes containing I_2_ 50 mM and different amounts of iodine salts; (B) computed equilibrium concentrations for electrolytes containing I^–^ 0.50 M + I_2_ 50 mM considering the reactions listed in Table S4.† Lines just connect data points for better reading and have no physical meaning.

Fig. 6A shows the intensity of the peaks attributed to I_3_^–^. The intensity of the peaks slightly increased over time, and these variations were higher than the standard deviation associated with absorbance values. Actually, it was found to be ±0.006 at 288 nm and ±0.005 at 350 nm (Table S3†). Licht *et al.* observed an enhancement of the I_3_^–^ signals intensity in acidified aqueous NaI + I_2_ solutions when compared to those with no added acid.^48^ The authors also observed the decrease of the I_3_^–^ signal and the gradual growth of a signal at 359 nm with increasing [I^–^] up to 10 M with I_2_ 10 mM. The authors attributed this signal to I_4_^2–^, though no such evidence was observed during our experiments for this species. On the other hand, in the case of aged electrolytes, acidic condition may be induced by CO_2_ dissolved in water. Moreover, the following reactions with dissolved O_2_ are likely to be involved:

7 O_2_ + 4H^+^ + 4e^–^ → 2H_2_O

8 4I^–^ → 4e^–^ + 2I_2_

where 
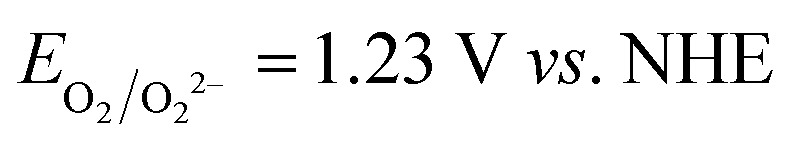
 and 
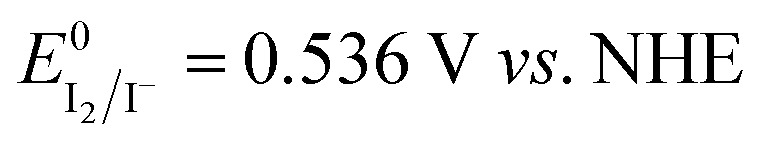
. I_2_ formed after this reaction is accounted to bind I^–^, resulting in the increase of I_3_^–^ concentration.

Besides the reaction leading to I_3_^–^ formation, at least nine additional equilibria involving I_2_ and I^–^ in aqueous medium have to be considered, including the association of two I_3_^–^ anions in I_6_^–^ and the formation of I_5_^–^. The other reactions follow from the formation of HIO. In these latter cases, the equilibrium concentrations depend upon the amount of added I^–^ relative to I_2_ and pH values. Specifically, the importance (from a quantitative point of view) of these species increases with decreasing the amount of added I^–^ and by increasing the pH value.

Iodate (IO_3_^–^) has been mentioned only a few times in the field of aqueous electrolytes. Macht *et al.* suggested that in the presence of water, I_2_ may undergo transformations over time, leading to the formation of IO_3_^–^, possibly in connection with the basic character of TBP.^12^ Therefore, the concentration of redox mediator in its oxidized form would decrease, thus leading to a decrease in FF as well. Choi *et al.* and Zhang *et al.*, to explain efficiency losses over time, also invoked the possible formation of IO_3_^–^.^27^,^46^ However, none of these authors clearly evidenced the actual presence of IO_3_^–^. In this work, the UV-vis spectra did not provide sufficiently detailed information about IO_3_^–^ presence in the electrolyte, therefore the conditions for its formation were evaluated by the model described in Section 2. In detail, IO_3_^–^ is formed in aqueous solution after the slow dismutation of HIO:

9 3HIO ↔ IO_3_^–^ + I^–^ + 3H^+^

If I^–^ is added to an aqueous I_2_ solution, [HIO] is very low under acidic and neutral conditions, so that IO_3_^–^ formation should be taken into account only above pH 7. At high I^–^ concentrations, the formation of HIO acid is negligible.^31^Fig. 6B displays the concentrations of iodine species in the presence of I^–^ 0.50 M and I_2_ 50 mM in the range 0 < pH < 14, which was computed based on the equilibrium constants for the reactions listed in Table S4.† Reliable pH values for a working DSSC are in the range of 2–8 to avoid dye desorption. Under these conditions, the maximum computed value of [HIO] is ≈10^–8^ M at I^–^ 0.50 M and I_2_ 50 mM (Fig. 6B and Table S1†). Considering that the formation of HIO and IO_3_^–^ is further inhibited at higher I^–^ concentrations, the formation of IO_3_^–^ is not likely to be the cause of the poor stability at the I^–^/I_2_ ratios commonly employed in aqueous DSSCs. As a matter of fact, Leandri *et al.* monitored the photovoltaic parameters of aqueous DSSCs for 20 days, at I^–^/I_2_ = 100/1 and pH = 8, and they observed an improvement of the cell performance over time.^21^ On the other hand, efficiency losses were recorded in the case of similar devices at pH = 9.

### 3.4 Electrochemical impedance spectroscopy analysis of aqueous solar cells

EIS spectra of DSSCs were recorded under dark conditions by applying to the photoanode a cathodic bias voltage corresponding to the *V*_oc_ value, previously measured under 1 sun irradiation.

**Fig. 7:**
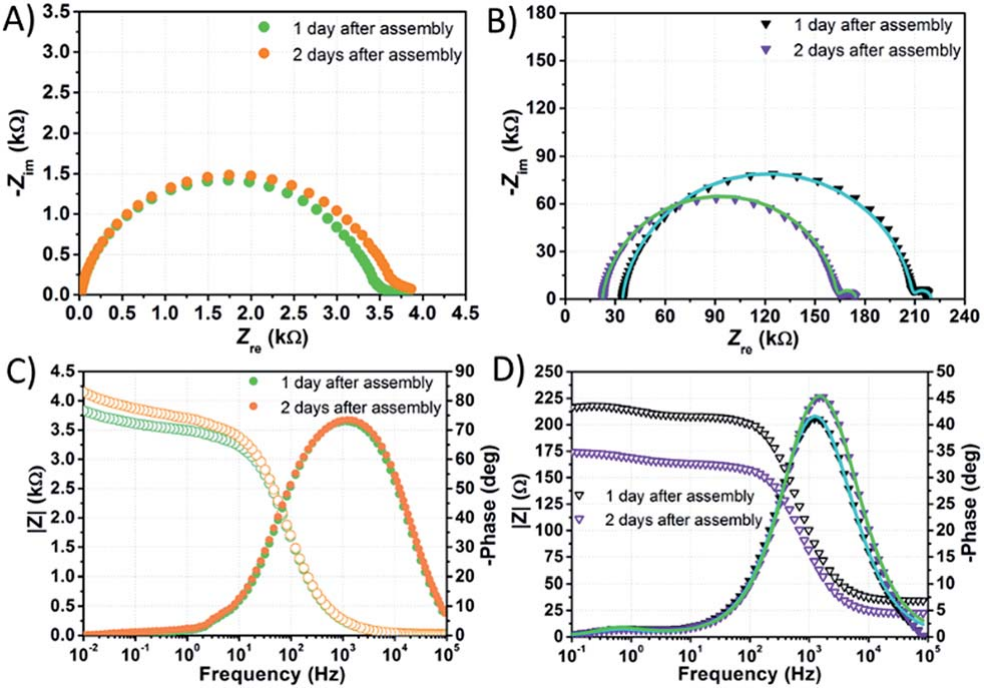
EIS spectra of aqueous DSSCs assembled with KI 1.50 M + I_2_ 50 mM (A and C) and NaI 1.50 M + I_2_ 50 mM (B and D). Data points were fitted (lines) according to eqn (3) with the constraints *R*_Pt_ = 2 Ω and *C*_Pt_ = 2 × 10^–5^ F.

In the Nyquist plot corresponding to the cell filled with NaI 1.50 M + I_2_ 50 mM (Fig. 7B and D), the response due to the processes at the counter-electrode is embedded in the arc due to charge accumulation and charge transfer at the TiO_2_/electrolyte interface. This feature is consistent with findings by Choi *et al.* related to water-based cells sensitized with organic JK262.^20^ Moreover, in this latter case, the signal due to I_3_^–^ diffusion is displayed as a small arc at high *Z*_re_ values. The signal due to I_3_^–^ diffusion matches pretty well with the response due to the same phenomenon in the Nyquist plot of the symmetric cell assembled using two Pt coated counter-electrodes and filled with NaI 1.50 M + I_2_ 50 mM. In the Bode plots of both samples the characteristic frequencies associated to I_3_^–^ diffusion are rather similar.

On the other hand, in the Nyquist plot of the cell filled with KI 1.50 M + I_2_ 50 mM (Fig. 7A) all the responses are embedded in one single signal; in the Bode plot, the peak due to I_3_^–^ diffusion is not clearly seen. In this case, resistances involved are strikingly high, and one or more of them must be higher by one order of magnitude if compared to those resulting from the NaI-based cell's Nyquist plot. High *R*_ct_ values may follow from poor wettability preventing the electron acceptors from approaching the recombination sites. Poor wetting of the photoanode would also explain the low values of *J*_sc_, FF and PCE at 1 sun recorded before EIS measurements, which were 0.31 mA cm^–2^, 0.47 and 0.06%, respectively. It is worth noting that the main peak in the Bode plot of KI-based cells (Fig. 7C) is larger than that displayed in the spectrum of its NaI counterpart (Fig. 7D), but the characteristic frequency is almost the same.

In the Nyquist plot of the KI-laden cell, the point at low *Z*_re_ values where the arc starts (namely, the series resistance *R*_s_) was found to decrease within the first two days by ≈4 Ω. Overall, *R*_s_ was found to decrease by 10 Ω and ≈4 Ω within the first two days in the case of NaI and KI, respectively, whereas it increased by ≈6 Ω for the EL-HSE-based cell (data not reported). This suggests that, in the presence of aqueous electrolytes, a sort of activation is required for the FTO layer as well.

**Fig. 8:**
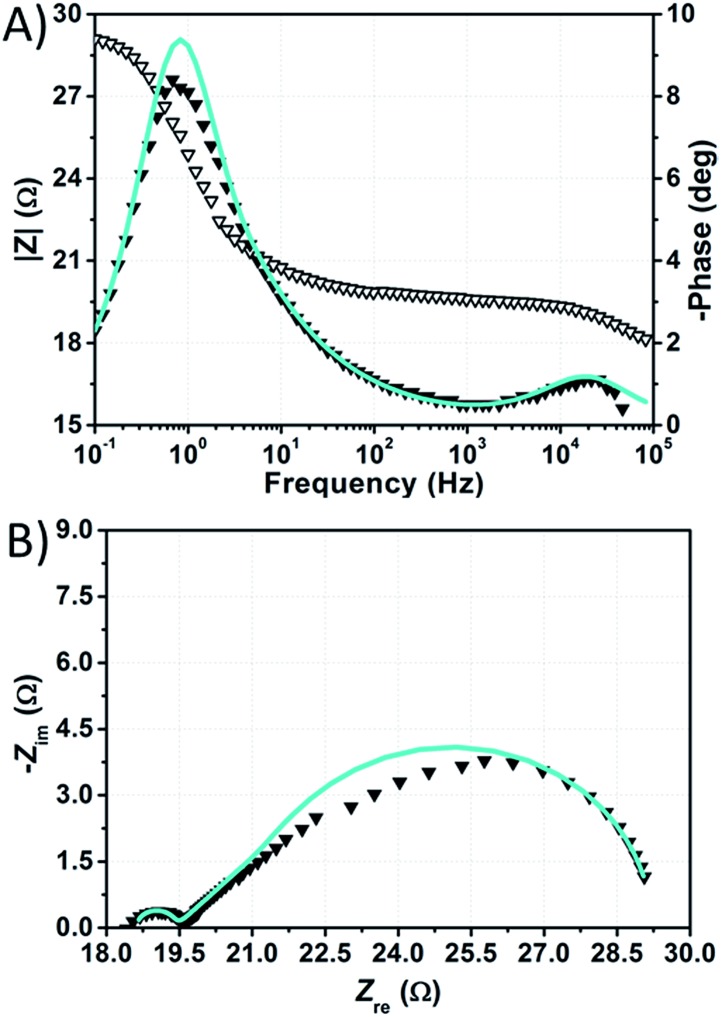
Phase (filled triangles) and modulus (open triangles) Bode (A) and Nyquist (B) plots of the dummy cell filled with aqueous electrolyte comprised of NaI 1.50 M + I_2_ 50 mM. Data points were fitted (light blue lines) according to eqn (1).

The impedance spectra of the dummy cell containing NaI 1.50 M and I_2_ 50 mM are shown in Fig. 8. The values of *R*_Pt_, *D* and *R*_d_ were found to be <1 Ω, 1.55 × 10^–5^ cm^2^ s^–1^ and ≈10 Ω, respectively (Table S5†). The values of *R*_Pt_ and *D* are similar to those reported by Hauch *et al.* for organic solvents-based electrolytes.^49^ Overall, none of these parameters was negatively affected by the aqueous electrolytes when compared to the common organic solvents-based ones.

The spectra of the NaI-based cell were fitted setting the parameters connected to the Pt/electrolyte interface at fixed values estimated from data related to the dummy cell. Actually, according to Hauch *et al.*,^49^ the applied potential does not induce large changes in the charge transfer resistance at the counter-electrode. In our system, *R*_Pt_ was arbitrary fixed at 2 Ω, whereas the capacitance at the Pt/electrolyte interface was computed from the CPE element of the symmetric cell and set to 2 × 10^–5^ F. In this way, the main signals in the impedance spectra of the NaI-based cell are almost completely ascribable to processes at the TiO_2_/electrolyte interface. Following this approach, data could be fitted resulting in *R*_ct_ values in the range of 130–170 Ω at the given applied potentials (Table S6†). It is worth noting that, in aqueous DSSCs, the recombination process seems to occur at the same characteristic frequency associated to charge transfer at the Pt/electrolyte interface in EL-HSE-based devices, namely ≈10^3^ Hz. Disabling the constraints on the counter-electrode parameters and fixing *T*_TiO_2__ at 10^–5^ or 10^–4^ Ω^–1^ s^φ^ resulted in high *R*_Pt_ values (>60 Ω) and effective electron lifetimes up to ≈1 ms (Tables S7 and S8†). Anyway, in these latter cases, the quality of the fitting was worse when compared to that obtained enabling the constraints on *R*_Pt_ and *C*_Pt_. On the one hand, the high *R*_Pt_ values would explain the poor performances of aqueous cells when compared to the reference EL-HSE. On the other hand, a question arises as to whether the *R*_Pt_ value can increase so much in DSSCs when compared to the value observed in the symmetric dummy cell. Further investigations are required for a thorough understanding, and will be the subject of a forthcoming article focused on EIS investigations at different potentials to better resolve the different signals. However, the D131/electrolyte interfacial characteristics can be derived also based on the findings by Jeanbourquin *et al.*,^37^ who showed that D131 (in its ground state) can interact strongly with I_2_. Therefore, it is reasonable to hypothesize that the formation of such complexes contributes to the fast recombination processes observed in aqueous DSSCs reported in this work. This effect may be enhanced even more during EIS measurements under dark conditions, as the dye remains in its ground state. Besides this, sizeable amounts of polyiodides, actually higher than I_3_^–^, are supposed to be present in all of the aqueous electrolytes prepared in this work (see Fig. 6B), but such species are not supposed to be involved in back-electron transfer.^50^

**Fig. 9:**
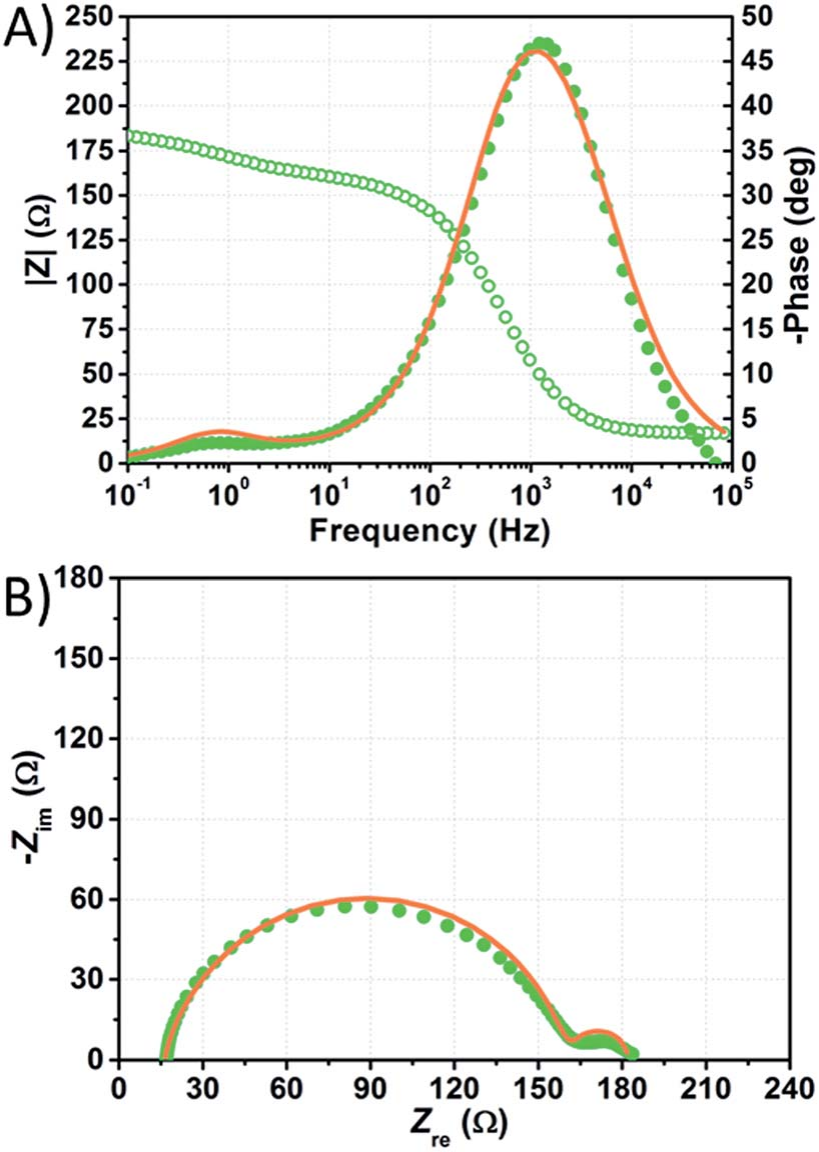
Phase (filled circles) and modulus (open circles) Bode (A) and Nyquist (B) plots of the dummy cell filled with aqueous electrolyte comprised of KI 1.50 M + I_2_ 50 mM. Data points, recorded five months after cell assembly, were fitted (lines) according to eqn (3) with the constraint *R*_Pt_ = 2 Ω and *C*_Pt_ = 2 × 10^–5^ F.

The impedance spectra of the most stable performing cell, which is filled with aqueous KI 5.50 M and I_2_ 50 mM, are shown in Fig. 9. In this case, *τ*_e_ is nearly doubled with respect to the value of electron lifetime of the NaI-based device for each constraint enabled during fitting (Table S8†). This fact suggests that KI-based cells are affected to a lesser extent by recombination, especially if one considers that the potential applied to these devices was more negative by over 100 mV. Nevertheless, *τ*_e_ is low when compared to that of the EL-HSE-filled cell or to the data reported by Zhang *et al.* for aqueous devices,^46^ suggesting that high dark current generation strongly affects the photovoltaic performance.^51^,^52^

Fig. 9 shows the impedance spectra of the KI-based cell fitted enabling the constraint *R*_Pt_ = 2 Ω and *C*_Pt_ = 2 × 10^–5^ F. Interestingly, in this case slightly better fitting curves were obtained disabling the constraints on *R*_Pt_ and *C*_Pt_; the resulting *R*_Pt_ and *R*_ct_ values were ≈40 and ≈100 Ω, respectively, under these conditions (Table S8†). Such values are much more reliable if compared to those obtained under the same conditions for the NaI-based device. Anyway, in the case of the KI-filled cells, the quality of the fitting curves was observed to be always lower when compared to the other cells (Table S8†).

Overall, EIS investigation on aqueous DSSCs provided three important features of aqueous electrolytes: (i) recombination at the photoanode/electrolyte interface could seem lower than that of standard electrolyte due to the inhibited electron acceptors approach to the recombination sites caused by the not perfect wettability of the electrode; (ii) an activation period is required for electrodes used in aqueous DSSCs; (iii) recombination phenomena are markedly influenced by the I^–^ counter-ion.

## 4. Conclusions

In this work the chemistry of iodine-based electrolytes for 100% aqueous DSSCs was thoroughly investigated. After an initial approach focused on the speciation of the various ionic and molecular species involved in the aqueous medium, the effect of the concentration of the I^–^ ions (responsible for the regeneration of the oxidized dye) was studied. Photocurrent was found to be the main factor affected by [I^–^]: the highest performance was measured in the presence of I^–^ 5.50 M. The effect of the counter-ion was also investigated: it emerged that KI salt exceeded the performance of NaI at high concentrations, reaching a PCE equal to ≈0.8% under 1 sun irradiation, in particular without using any additives or specific treatments of the photoanode surface. In all the other (lower) concentrations explored, the photovoltaic performances of NaI-based cells overtook their KI counterparts. Finally, the effect of I_2_ content was studied: enhanced photovoltage values were recorded in the presence of low [I_2_], due to reduced back-electron transfer reactions. However, these cells showed lower photocurrent values, thus leading overall to lower PCE values because of mass diffusion limitations.

Since the stability of aqueous DSSCs represents a big issue nowadays, we demonstrated that the PCE of our 100% aqueous devices remained perfectly stable during a five months aging test, without using any kind of stabilizers (*i.e.*, polymer, volatility suppressant, *etc.*). Besides this, by measuring EIS spectra of dummy and complete cells, a good similarity in terms of counter-electrode/electrolyte interface between standard and aqueous DSSCs emerged. On the other hand, lower electron lifetimes were measured for aqueous cells, thus suggesting that high dark current generation is one of the main factors affecting the photovoltaic performances of these emerging devices. For the present D131 dye-based system, such a behaviour is most likely ascribable to the formation of dye–I_2_ complexes, allowing for high concentrations of electron acceptors for back electron transfer around the semiconductor. Moreover, the reasonable vertical orientation of this dye molecule with respect to the semiconductor surface may allow for the fast diffusion of the acceptor species in the electrolyte towards the recombination sites. Further investigations about the specific interactions of different dye molecules with iodine-based electrolytes in aqueous media, together with redox couple entrapment in polymeric matrices,^53^–^55^ are worthy of investigation, and are at present the object of intense research activity in our labs.

## Supplementary Material

Supplementary information

## References

[cit1] Bella F., Gerbaldi C., Barolo C., Grätzel M. (2015). Chem. Soc. Rev..

[cit2] Ying W., Yang J., Wielopolski M., Moehl T., Moser J. E., Comte P., Hua J., Zakeeruddin S. M., Tian H., Grätzel M. (2014). Chem. Sci..

[cit3] Wang S. W., Wu K. L., Ghadiri E., Lobello M. G., Ho S. T., Chi Y., Moser J. E., De Angelis F., Grätzel M., Nazeeruddin M. K. (2013). Chem. Sci..

[cit4] Aribia K. B., Moehl T., Zakeeruddin S. M., Grätzel M. (2013). Chem. Sci..

[cit5] Liu P., Gardner J. M., Kloo L. (2015). Chem. Commun..

[cit6] Liu Y., Hagfeldt A., Xiao X. R., Lindquist S. E. (1998). Sol. Energy Mater. Sol. Cells.

[cit7] Weidmann J., Dittrich T., Konstantinova E., Lauermann I., Uhlendorf I., Koch F. (1999). Sol. Energy Mater. Sol. Cells.

[cit8] De Angelis F., Fantacci S., Gebauer R. (2011). J. Phys. Chem. Lett..

[cit9] Sumita M., Sodeyama K., Han L., Tateyama Y. (2011). J. Phys. Chem. C.

[cit10] Agrell H. G., Lindgren J., Hagfeldt A. (2003). Sol. Energy.

[cit11] Lu H. L., Shen T. F. R., Huang S. T., Tung Y. L., Yang T. C. K. (2011). Sol. Energy Mater. Sol. Cells.

[cit12] Macht B., Turrion M., Barkschat A., Salvador P., Ellmer K., Tributsch H. (2002). Sol. Energy Mater. Sol. Cells.

[cit13] Kitamura T., Okada K., Matsui H., Tanabe N. (2010). J. Sol. Energy Eng..

[cit14] Dong C., Xiang W., Huang F., Fu D., Huang W., Bach U., Cheng Y. B., Li X., Spiccia L. (2014). Angew. Chem., Int. Ed..

[cit15] Law C., Moudam O., Villarroya-Lidon S., O'Regan B. C. (2012). J. Mater. Chem..

[cit16] Kato R., Kato F., Oyaizu K., Nishide H. (2014). Chem. Lett..

[cit17] Tian H., Gabrielsson E., Lohse P. W., Vlachopoulos N., Kloo L., Hagfeldt A., Sun L. (2012). Energy Environ. Sci..

[cit18] Daeneke T., Uemura Y., Duffy N. W., Mozer A. J., Koumura N., Bach U., Spiccia L. (2012). Adv. Mater..

[cit19] Xiang W., Chen D., Caruso R. A., Cheng Y. B., Bach U., Spiccia L. (2015). ChemSusChem.

[cit20] Choi H., Jeong B. S., Do K., Ju M. J., Song K., Ko J. (2013). New J. Chem..

[cit21] Leandri V., Ellis H., Gabrielsson E., Sun L., Boschloo G., Hagfeldt A. (2014). Phys. Chem. Chem. Phys..

[cit22] Lin R. Y. Y., Chuang T. M., Wu F. L., Chen P. Y., Chu T. C., Ni J. S., Fan M. S., Lo Y. H., Ho K. C., Lin J. T. (2015). ChemSusChem.

[cit23] Xiang W., Huang F., Cheng Y. B., Bach U., Spiccia L. (2013). Energy Environ. Sci..

[cit24] Saito H., Uegusa S., Murakami T. N., Kawashima N., Miyasaka T. (2004). Electrochemistry.

[cit25] Jung Y. S., Yoo B., Lim M. K., Lee S. Y., Kim K. J. (2009). Electrochim. Acta.

[cit26] Murakami T. N., Saito H., Uegusa S., Kawashima N., Miyasaka T. (2003). Chem. Lett..

[cit27] Choi H., Han J., Kang M. S., Song K., Ko J. (2014). Bull. Korean Chem. Soc..

[cit28] Park S. J., Yoo K., Kim J. Y., Kim J. Y., Lee D. W., Kim B., Kim H., Kim J. H., Cho J., Ko M. J. (2013). ACS Nano.

[cit29] Koenigsmann C., Ripolles T. S., Brennan B. J., Negre C. F. A., Koepf M., Durrell A. C., Milot R. L., Torre J. A., Crabtree R. H., Batista V. S., Brudvig G. W., Bisquert J., Schmuttenmaer C. A. (2014). Phys. Chem. Chem. Phys..

[cit30] Su Y. H., Lai W. H., Teoh L. G., Hon M. H., Huang J. L. (2007). Appl. Phys. A: Mater. Sci. Process..

[cit31] Gottardi W. (1999). Arch. Pharm..

[cit32] Horiuchi T., Miura H., Uchida S. (2004). J. Photochem. Photobiol., A.

[cit33] Tagliaferro R., Gentilini D., Mastroianni S., Zampetti A., Gagliardi A., Brown T. M., Reale A., Di Carlo A. (2013). RSC Adv..

[cit34] Macdonald J. and Barsoukov E., Impedance spectroscopy: theory experiment and applications, John Wiley & Sons, Hoboken, New Jersey, 2005.

[cit35] Hagfeldt A., Boschloo G., Sun L., Kloo L., Pettersson H. (2010). Chem. Rev..

[cit36] Kusama H., Sugihara H., Sayama K. (2011). J. Phys. Chem. C.

[cit37] Jeanbourquin X., Li X., Law C., Barnes P., Humphry-Baker R., Lund P., Asghar M. I., O'Regan B. C. (2014). J. Am. Chem. Soc..

[cit38] Goldsack D., Franchetto R. (1977). Can. J. Chem..

[cit39] Marcus Y. (2010). J. Chem. Eng. Data.

[cit40] Wang Y. (2009). Sol. Energy Mater. Sol. Cells.

[cit41] Boschloo G., Hagfeldt A. (2009). Acc. Chem. Res..

[cit42] Galliano S., Bella F., Gerbaldi C., Falco M., Viscardi G., Grätzel M. and Barolo C., Photoanode/Electrolyte Interface Stability in Aqueous Dye-Sensitized Solar Cells, under review. 10.1039/c6sc01145d PMC6014110 30155136

[cit43] Chen C., Yang X., Cheng M., Zhang F., Sun L. (2013). ChemSusChem.

[cit44] Soni S. S., Fadadu K. B., Vekariya R. L., Debgupta J., Patel K. D., Gibaud A., Aswal V. K. (2014). J. Colloid Interface Sci..

[cit45] Dai Q., Rabani J. (2001). Chem. Commun..

[cit46] Zhang H., Qiu L., Xu D., Zhang W., Yan F. (2014). J. Mater. Chem. A.

[cit47] Awtrey A., Connick R. (1951). J. Am. Chem. Soc..

[cit48] Licht S., Myung N. (1995). J. Electrochem. Soc..

[cit49] Hauch A., Georg A. (2001). Electrochim. Acta.

[cit50] Rowley J., Farnum B., Ardo S., Meyer G. (2010). J. Phys. Chem. Lett..

[cit51] Bella F., Lamberti A., Sacco A., Bianco S., Chiodoni A., Bongiovanni R. (2014). J. Membr. Sci..

[cit52] Wee K. R., Sherman B. D., Brennaman M. K., Sheridan M. V., Nayak A., Alibabaei L., Meyer T. J. (2016). J. Mater. Chem. A.

[cit53] Sun B., Xu C., Mindemark J., Gustafsson T., Edström K., Brandell D. (2015). J. Mater. Chem. A.

[cit54] Costa L. T., Sun B., Jeschull F., Brandell D. (2015). J. Chem. Phys..

[cit55] Raja M., Angulakshmi N., Thomas S., Kumar T. P., Stephan A. M. (2014). J. Membr. Sci..

