# Supplementary material for: Unveiling iodine-based electrolytes chemistry in aqueous dye-sensitized solar cells
Source: Chem Sci. 2016 Apr 13;7(8):4880–90. doi: 10.1039/c6sc01145d (PMC6014110; doi:10.1039/c6sc01145d)
Supplement: Supplementary file 1 [file SC-007-C6SC01145D-s001.pdf]

Electronic Supplementary Material (ESI) for Chemical Science.
This journal is © The Royal Society of Chemistry 2016

Unveiling iodine-based electrolytes chemistry in aqueous dye-sensitized solar cells

Federico **Bella**,[1,*] Simone **Galliano**,[2] Marisa **Falco**,[2] Guido **Viscardi**,[2] Claudia **Barolo**[2,*], Michael **Grätzel**,[3] Claudio **Gerbaldi**[1,*]

1) *GAME Lab, CHENERGY Group, Department of Applied Science and Technology - DISAT, Politecnico di Torino, Corso Duca degli Abruzzi 24, 10129 – Torino, Italy*
2) *Department of Chemistry and NIS Interdepartmental Centre, Università degli Studi di Torino, Via Pietro Giuria 7, 10125 - Torino, Italy*
3) *Laboratory of Photonics and Interfaces, Institut des Sciences et Ingénierie Chimiques, Ecole Polytechnique Fédérale de Lausanne (EPFL), Station 3, CH1015 - Lausanne, Switzerland*

SUPPORTING INFORMATION

Table S1. Computed equilibrium concentrations (in M = mol L^{-1}) of iodine compounds in aqueous electrolyte composed of I^- 0.50 M and I_2 50 mM. Details about the speciation model are given in **Appendix 1**.

**pH**	**I_2**	**I^-**	**HIO**	**IO^-**	**HI_2O^-**	**I_3^-**	**I_5^-**	**I_6^{2-}**	**I_2O^{2-}**	**|Δ|**
0	1.5×10^{-04}	4.5×10^{-01}	2.3×10^{-16}	6.6×10^{-27}	3.1×10^{-14}	4.9×10^{-02}	6.7×10^{-05}	2.3×10^{-04}	1.2×10^{-28}	1.2×10^{-05}
1	1.5×10^{-04}	4.5×10^{-01}	2.3×10^{-15}	6.6×10^{-25}	3.1×10^{-13}	4.9×10^{-02}	6.7×10^{-05}	2.3×10^{-04}	1.2×10^{-26}	1.2×10^{-05}
2	1.5×10^{-04}	4.5×10^{-01}	2.3×10^{-14}	6.6×10^{-23}	3.1×10^{-12}	4.9×10^{-02}	6.7×10^{-05}	2.3×10^{-04}	1.2×10^{-24}	1.2×10^{-05}
3	1.5×10^{-04}	4.5×10^{-01}	2.3×10^{-13}	6.6×10^{-21}	3.1×10^{-11}	4.9×10^{-02}	6.7×10^{-05}	2.3×10^{-04}	1.2×10^{-22}	1.2×10^{-05}
4	1.5×10^{-04}	4.5×10^{-01}	2.3×10^{-12}	6.6×10^{-19}	3.1×10^{-10}	4.9×10^{-02}	6.7×10^{-05}	2.3×10^{-04}	1.2×10^{-20}	1.2×10^{-05}
5	1.5×10^{-04}	4.5×10^{-01}	2.3×10^{-11}	6.6×10^{-17}	3.1×10^{-09}	4.9×10^{-02}	6.7×10^{-05}	2.3×10^{-04}	1.2×10^{-18}	1.2×10^{-05}
6	1.5×10^{-04}	4.5×10^{-01}	2.3×10^{-10}	6.6×10^{-15}	3.1×10^{-08}	4.9×10^{-02}	6.7×10^{-05}	2.3×10^{-04}	1.2×10^{-16}	1.2×10^{-05}
7	1.5×10^{-04}	4.5×10^{-01}	2.3×10^{-09}	6.6×10^{-13}	3.1×10^{-07}	4.9×10^{-02}	6.7×10^{-05}	2.3×10^{-04}	1.2×10^{-14}	1.2×10^{-05}
8	1.5×10^{-04}	4.5×10^{-01}	2.3×10^{-08}	6.6×10^{-11}	3.1×10^{-06}	4.9×10^{-02}	6.7×10^{-05}	2.3×10^{-04}	1.2×10^{-12}	1.2×10^{-05}
9	1.5×10^{-04}	4.5×10^{-01}	2.3×10^{-07}	6.6×10^{-09}	3.1×10^{-05}	4.9×10^{-02}	6.7×10^{-05}	2.3×10^{-04}	1.2×10^{-10}	1.3×10^{-05}
10	1.5×10^{-04}	4.5×10^{-01}	2.3×10^{-06}	6.6×10^{-07}	3.1×10^{-04}	4.9×10^{-02}	6.6×10^{-05}	2.3×10^{-04}	1.2×10^{-08}	1.1×10^{-05}
11	1.4×10^{-04}	4.5×10^{-01}	2.1×10^{-05}	6.1×10^{-05}	2.9×10^{-03}	4.6×10^{-02}	5.9×10^{-05}	2.1×10^{-04}	1.2×10^{-06}	1.1×10^{-05}
12	8.4×10^{-05}	4.8×10^{-01}	1.2×10^{-04}	3.5×10^{-03}	1.7×10^{-02}	2.9×10^{-02}	2.2×10^{-05}	8.0×10^{-05}	6.9×10^{-05}	2.3×10^{-05}
13	7.6×10^{-06}	5.3×10^{-01}	1.0×10^{-04}	3.0×10^{-02}	1.6×10^{-02}	2.9×10^{-03}	2.0×10^{-07}	8.1×10^{-07}	6.7×10^{-04}	1.2×10^{-05}
14	1.2×10^{-07}	5.5×10^{-01}	1.5×10^{-05}	4.6×10^{-02}	2.5×10^{-03}	4.6×10^{-05}	4.7×10^{-11}	2.0×10^{-10}	1.1×10^{-03}	1.2×10^{-05}

Table S2. Intensities of I_3^- signals of diluted aqueous solutions obtained from the aqueous electrolytes containing different amounts of NaI or KI salts (in M = mol L^{-1}) at different times after the preparation of stock solutions. Amount of I_2 was fixed at 50 mM.

**Stock solution**	**Abs (288 nm) of diluted solutions**			
**$[I^-]$**	**KI-5th day**	**NaI-6th day**	**KI-12th day**	**NaI-13th day**
0.50	1.55721	1.52340	1.42963	1.54030
1.50	2.62827	2.60157	2.69459	2.63125
2.50	3.00280	3.00370	2.99818	3.05316

**Stock solution**	**Abs (350 nm) of diluted solutions**			
**$[I^-]$**	**KI-5th day**	**NaI-6th day**	**KI-12th day**	**NaI-13th day**
0.50	1.02763	0.99950	0.93885	1.01544
1.50	1.72065	1.70602	1.77037	1.72044
2.50	1.98118	1.97521	1.97418	2.01815

**Stock solution**	**Abs (288 nm) of diluted solutions**					
**$[I^-]$**	**KI-fresh**	**NaI-fresh**	**KI-12th day**	**NaI-13th day**	**KI-22th day**	**NaI-22th day**
0.50			0.40829	0.36113	0.45156	0.47799
1.50			0.98442	0.98268	0.98899	1.04906
2.50			1.24581	1.22466	1.21360	1.21444
3.50	1.38951	1.36403	1.46602	1.43965	1.47703	1.46814
4.50	1.51819	1.47913	1.58296	1.55105	1.58003	1.57745
5.50	1.54363	1.53191	1.56974	1.51077	1.60015	1.53093
6.50		1.67016		1.57254		1.70860

**Stock solution**	**Abs (350 nm) of diluted solutions**					
**$[I^-]$**	**K-fresh**	**Na-fresh**	**K-12th day**	**Na-13th day**	**K-22th day**	**Na-22th day**
0.50			0.27737	0.24738	0.29812	0.30927
1.50			0.65846	0.65933	0.65511	0.69171
2.50			0.83086	0.80328	0.79574	0.79854
3.50	0.91226	0.90244	0.96057	0.95323	0.96296	0.96021
4.50	0.99916	0.97519	1.03695	1.01879	1.03844	1.04028
5.50	1.01542	1.01878	1.02871	0.99604	1.05670	1.00843
6.50		1.09712		1.03322		1.12200

Table S3. Standard deviations associated to the maximum absorption wavelengths (in nm) and to the signals intensities of I_3^-. 1:1000 diluted solutions were prepared from NaI 0.50 M + I_2 25 mM.

Sample	λ_1	Abs (λ_1)	λ_2	Abs (λ_2)
**1**	288.0	0.20975	350.0	0.14769
**2**	288.0	0.21191	350.0	0.1485
**3**	287.0	0.19843	350.0	0.13638
**4**	287.5	0.21435	350.0	0.15027
**5**	289.0	0.20439	350.5	0.14275
**6**	288.0	0.20788	350.0	0.14385
**average**	287.9	0.20779	350.1	0.14491
**sd**	0.7	5.71×10^{-03}	0.2	5.06×10^{-03}

Table S4. Equilibria involving aqueous solutions containing I_2 and I^-.

$K_1 = 5.44\times10^{-14}$ M^{-1}	$H_2O + I_2 \leftrightarrow HIO + I^- + H^+$
$K_2 = 2.3\times10^{-11}$ M^{-1}	$HIO \leftrightarrow IO^- + H^+$
$K_3 = 723$ M^{-1}	$I^- + I_2 \leftrightarrow I_3^-$
$K_5 = 9$ M^{-1}	$I_3^- + I_2 \leftrightarrow I_5^-$
$K_6 = 0.1$ M^{-1}	$2I_3^- \leftrightarrow I_6^{2-}$
$K_7 = 0.13$ M^{-1}	$IO^- + I^- + H_2O \leftrightarrow HI_2O^- + OH^-$

$K_8 = 0.35\times10^{-15}$ M^{-1}	$HI_2O^- \leftrightarrow I_2O^{2-} + H^+$

Table S5. Parameters obtained by fitting impedance spectra of the dummy cell filled with aqueous NaI 1.5 M + I_2 50 mM.

**Symmetric cell**								**Single electrode**	
**2*R*$_s$ (Ω)**	**2*R*$_{Pt}$ (Ω)**	***R*$_d$ (Ω)**	***T*$_{Pt}$ (Ω^{-1} s^{φ})**	**$φ_{Pt}$**	***D* (cm^2 s^{-1})**	**½ *C*$_{Pt}$ (F)**	**WSS (Ω)**	***R*$_{Pt}$ (Ω)**	***C*$_{Pt}$ (F)**
18.6	0.8	9.8	1.52×10^{-05}	0.97	1.55×10^{-05}	1.01×10^{-05}	0.38	0.4	2.02×10^{-05}

Table S6. Parameters obtained by fitting the impedance spectra of aqueous DSSCs with the constraints: $R_{Pt} = 2$ Ω, $C_{Pt} = 2 \times 10^{-5}$ F.

**Cell**	***R*$_s$ (Ω)**	***R*$_{Pt}$ (Ω)**	**T_{TiO_2} (Ω^{-1} s^{φ})**	**$φ_{TiO2}$**	***C*$_{Pt}$ (F)**	***R*$_{ct}$ (Ω)**	***R*$_d$ (Ω)**	***D* (cm^2 s^{-1})**	**$τ_e$ (ms)**	***C*$_μ$ (F)**	**WSS (Ω)**
**NaI-based 24 h**	34.42	2	2.96×10^{-06}	0.95	2.00×10^{-05}	171.26	12.11	1.44×10^{-05}	0.33	1.91×10^{-06}	0.76
**NaI-based 24 h**	22.40	2	3.09×10^{-06}	0.96	2.00×10^{-05}	138.01	12.30	1.44×10^{-05}	0.30	2.16×10^{-06}	0.52
**KI-based 156 d**	16.40	2	7.63×10^{-06}	0.90	2.00×10^{-05}	139.61	24.03	1.62×10^{-05}	0.50	3.56×10^{-06}	3.59

Table S7. Parameters obtained by fitting the impedance spectra of aqueous DSSCs with the constraint: $T_{TiO_2} \geq 10^{-5}$ Ω^{-1} s^{φ}.

**Sample**	***R*$_s$ (Ω)**	***R*$_{Pt}$ (Ω)**	**T_{TiO_2} (Ω^{-1} s^{φ})**	**$φ_{TiO2}$**	***C*$_{Pt}$ (F)**	***R*$_{ct}$ (Ω)**	***R*$_d$ (Ω)**	***D* (cm^2 s^{-1})**	**$τ_e$ (ms)**	***C*$_μ$ (F)**	**WSS (Ω)**
**NaI-based 24 h**	34.86	81.03	1.04×10^{-05}	0.90	2.96×10^{-06}	92.90	9.24	1.38×10^{-05}	0.45	4.82×10^{-06}	1.50
**NaI-based**	22.53	65.72	1.04×10^{-05}	0.91	3.57×10^{-06}	75.92	8.77	1.44×10^{-05}	0.39	5.08×10^{-06}	0.70

**24 h**											
**KI-based 156 d**	16.99	39.99	1.37×10^{-05}	0.90	5.85×10^{-06}	101.81	23.00	1.62×10^{-05}	0.67	6.62×10^{-06}	1.80

Table S8. Parameters obtained by fitting the impedance spectra of aqueous DSSCs with the constraint: $T_{TiO_2} \geq 10^{-4}\ \Omega^{-1}\ s^{\varphi}$.

Sample	R_s (Ω)	R_{Pt} (Ω)	T_{TiO_2} (Ω^{-1} s^{φ})	φ_{TiO2}	C_{Pt} (F)	R_{ct} (Ω)	R_d (Ω)	D (cm^2 s^{-1})	τ_e (ms)	C_{μ} (F)	WSS (Ω)
**NaI-based 24 h**	35.55	149.30	1.00×10^{-04}	0.90	1.87×10^{-06}	23.49	11.57	1.44×10^{-05}	1.20	5.10×10^{-05}	3.80
**NaI-based 24 h**	23.14	133.10	3.76×10^{-04}	0.92	2.09×10^{-06}	4.42	15.46	1.44×10^{-05}	0.94	2.12×10^{-04}	4.00
**KI-based 156 d**	17.64	104.67	1.00×10^{-04}	0.90	3.23×10^{-06}	38.73	20.40	1.62×10^{-05}	2.09	5.40×10^{-05}	3.40

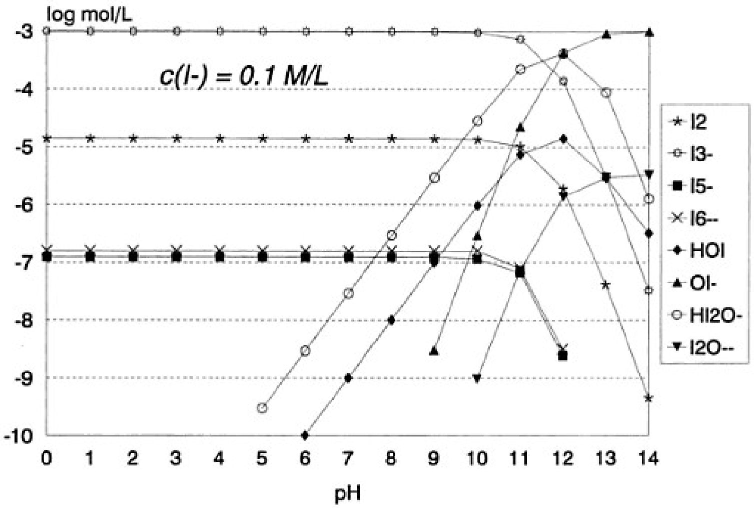

Figure S1. Computed Log*C* of iodine compounds in a solution containing aqueous I^- 0.10 M and I_2 1.0 mM as a function of pH. Data obtained by Gottardi and reprinted from [1] with permission. Lines just connect data points for better reading and have no physical meaning.

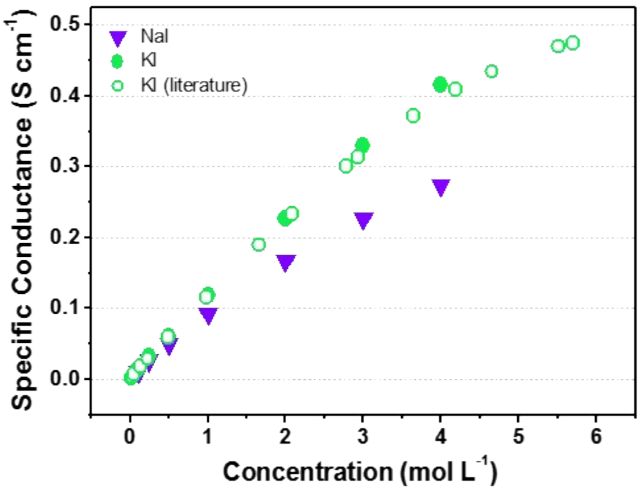

Figure S2. Specific conductance of aqueous NaI and KI solutions resulting from the equivalent conductance data at different I^- concentrations extracted from reference [2]. More recent data (from reference [3]) referred to aqueous KI solutions are shown for comparison.

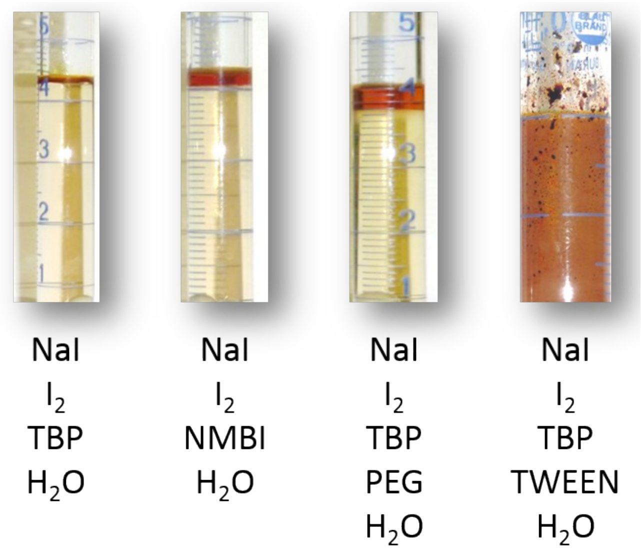

Figure S3. NaI/I_2 aqueous mixtures with different V_{oc} enhancers (TBP, NMBI) and surfactants (i.e., PEG300, TWEEN).

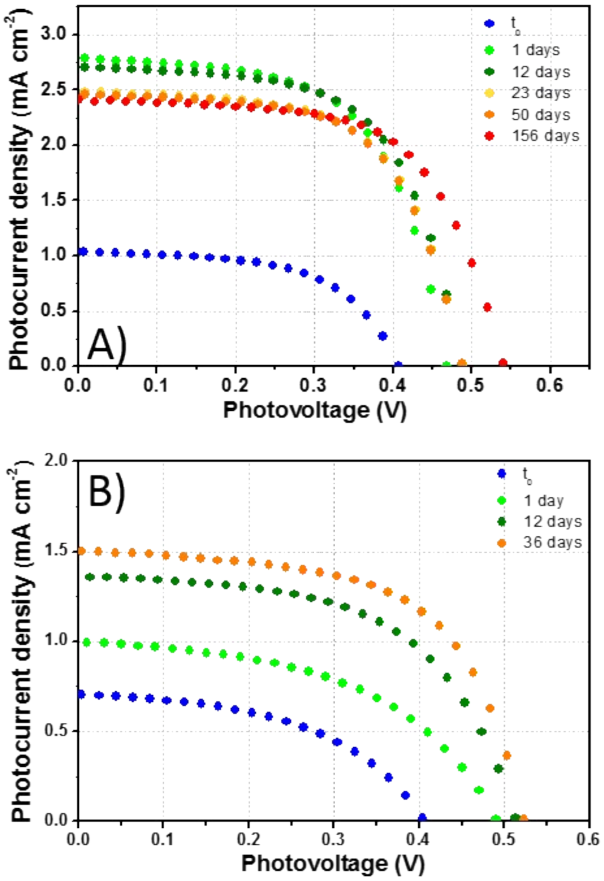

Figure S4. J-V curves of the most performing long lasting aqueous DSSCs: A) KI 5.50 M + I_2 50 mM; B) NaI 2.75 M + I_2 25 mM.

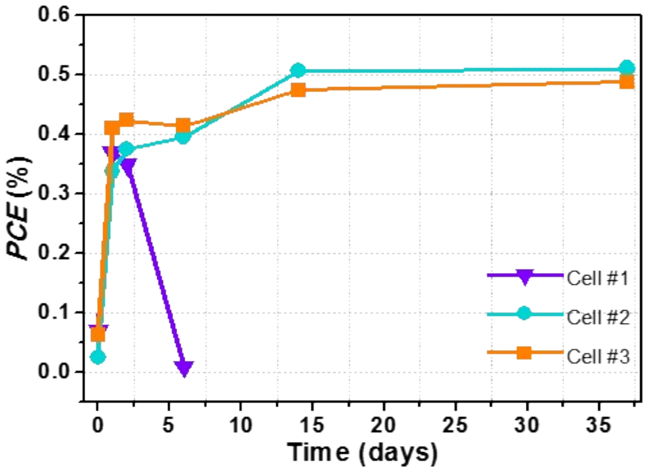

Figure S5. *PCE* values upon time of three DSSCs filled with aqueous NaI 1.50 M + I_2 50 mM. Devices #2 and #3 were sealed after drying the entry slit to avoid any possible contamination of the electrolyte by glue. Lines just connect data points and have no physical meaning.

Appendix 1 – Determination of iodine species in aqueous electrolytes

The concentrations of I_3^- and I^- are usually calculated from **Eq. 2** considering the equilibrium in **Eq. 1**:

$$I_3^- + 2e^- \leftrightarrow 3I^- \quad \text{(Eq. 1)}$$

$$K = \frac{[I_3^-]}{([I^-]_0 - [I_3^-])([I_2]_0 - [I_3^-])} \quad \text{(Eq. 2)}$$

where K = 723 L mol^{-1} in H_2O and $K = 5.8 \times 10^6$ L mol^{-1} in acetonitrile [4,5]. Therefore, in this work the equilibrium concentrations of I_3^-, I^- and I_2 were calculated according to **Eq. 2** assuming that the activity equilibrium constant equals K = 723 L mol^{-1} at 25 °C and that the activity coefficients ratio is equal to one. The literature value of K we used was determined spectrophotometrically at low I^- and I_2 concentrations [6]. Palmer *et al.* found that the values of I_2 activity coefficient (γ_{I_2}) and the ratio ($\gamma_{I_3^-}/\gamma_{I^-}$) vary by about the same quantities at ionic strengths up to ≈6 in perchlorate salt: data suggest that the value of ($\gamma_{I_3^-}/\gamma_{I^-}\gamma_{I_2}$) ratio should be equal to ≈1 over the investigated range [7].

Equilibrium concentrations of I_3^- and I^- obtained as described above were used to calculate the redox potential of the electrolytes according to the Nernst equation:

$$E_{redox} = E^0(I_3^-/I^-) + \frac{RT}{2F} ln \frac{[I_3^-]}{[I^-]^3} \quad \text{(Eq. 3)}$$

assuming $E^0(I_3^-/I^-)$ = 0.536 V [4]. In this case, the use of the standard potential instead of the formal one may result in sizeable errors. Taking as references the mean activity coefficients $\gamma_{\pm I_3^-}$ and $\gamma_{\pm I^-}$ obtained by Palmer *et al.* at ionic strengths up to ≈6 in $NaClO_4$ [7], errors up to 10 mV may be expected. The activity coefficients of I^- in highly concentrated

solutions would be required for accurate estimations. Pitzer parameters (e.g., parameters useful to understand the behavior of ions dissolved in water [8]) are compiled for NaI and KI solutions, but the expression returning the mean activity coefficients requires the value of ionic strength, which is affected by the formation of ion pairs. The evaluation of the activity coefficient of I_2 in salt solutions is often extrapolated from solubility data [9]. Such data are available in a wide range of I^- concentrations, but the formation of complexes does not allow to use the salting-out approximation.

For electrolytes containing I^- 0.50 M and I_2 50 mM, the equilibrium concentrations of higher polyiodides and the species following from the formation of hypoiodous acid (HIO) were computed on the basis of the algorithm developed by Gottardi [5]. The activity coefficient of uncharged species was set to equal one, and the activity coefficient of charged species was calculated by Debye-Hückel equation with an additional empirical term. Such assumptions allowed the author to obtain good agreement between the experimentally determined concentrations and the calculated ones (± ≈2% for potentiometric/titrimetric determinations and ± ≈5% for photometric measurements) up to I^- ≈0.60 M [10].

The equilibrium concentrations of iodine species were computed as a function of the pH. At I^- concentrations higher than 0.50 M, the Debye-Hückel approximation is not suitable to calculate the activity coefficients of the charged species and sizeable errors are expected assuming the activity coefficient of uncharged species such as HIO and I_2 equals to one.

Therefore, we propose the following mass balances:

$$C = [I_2] + [I_3^-] + 2[I_5^-] + 2[I_6^{2-}] + [HIO] + [IO^-] + [HI_2O^-] = Added\ iodine \quad \text{(Eq. 4)}$$

$$D = [I^-] + [I_3^-] + [I_5^-] + 2[I_6^{2-}] - [HIO] - [IO^-] = Added\ iodide \quad \text{(Eq. 5)}$$

$$T = 2C + D = Total\ iodine\ atoms \quad \text{(Eq. 6)}$$

The molar concentrations of the species listed above can be expressed through the following equations:

$$[HIO] = \frac{K_1\,[I_2]}{[H^+][I^-]\,fa} = \frac{BP\,[I_2]}{[I^-]\,fa} \quad \text{(Eq. 7)}$$

$$[IO^-] = \frac{K_2\,[HIO]}{[H^+][I^-]\,fa} = \frac{K_2\,K_1\,[I_2]}{[H^+]^2[I^-]\,fa^2} = \frac{BR\,[I_2]}{[I^-]\,fa^2} \quad \text{(Eq. 8)}$$

$$[I_3^-] = K_3\,[I_2][I^-] \quad \text{(Eq. 9)}$$

$$[I_6^{2-}] = \frac{K_6\,[I_3^-]^2\,fa^2}{ft} = \frac{K_6\,K_3\,[I_2]^2[I^-]^2\,fa^2}{ft} = \frac{P\,[I_2]^2[I^-]^2\,fa^2}{ft} \quad \text{(Eq. 10)}$$

$$[I_5^-] = K_5\,[I_3^-][I_2] = K_5\,K_3\,[I_2]^2[I^-] = N\,x_1^2\,x_2 \quad \text{(Eq. 11)}$$

$$[HI_2O^-] = \frac{K_7\,[I^-][IO^-]\,fa}{[OH^-]} = \frac{K_7\,10^{14}\,K_2\,K_1\,[I_2]}{[H^+]\,fa} = \frac{QQ\,x_1}{fa} \quad \text{(Eq. 12)}$$

$$[I_2O^{2-}] = \frac{K_8\,[HI_2O^-]\,fa}{[H^+]\,ft} = \frac{K_8\,K_7\,10^{14}\,K_2\,K_1\,[I_2]}{[H^+]^2\,ft} = \frac{QJ\,x_1}{ft} \quad \text{(Eq. 13)}$$

where the various constants and the concentration of hydrogen ions $[H^+]$ are incorporated in *BP*, *BR*, *QQ* and *QJ*; *fa* is the activity coefficient of singly charged species (assumed to be the same for each of them), *ft* is the activity coefficient of doubly charged species (assumed to be the same for each of them), $K_1 = 5.44 \times 10^{-13}$, $K_2 = 2.3 \times 10^{-11}$, $K_3 = 723$, $K_5 = 9$, $K_6 = 0.1$, $K_7 = 0.13$, $K_8 = 3.3 \times 10^{-15}$.

The activity coefficients were computed as follows:

$$-\log\gamma = \frac{0.509\,e^2\,\mu^2}{1 + 0.328\,d\,\sqrt{\mu}} + 0.3\mu \quad \text{(Eq. 14)}$$

where $e = 1$ and $d = 3$ for singly charged species, or $e = 2$ and $d = 4$ for doubly charged species. The ionic strength μ is given by:

$$\mu = [I^-] + [I_3^-] + [I_5^-] + [IO^-] + 3[I_6^{2-}] + 3[HI_2O^-] \quad \text{(Eq. 15)}$$

The following equations with variables x_1 = [I_2], x_2 = [I^-] and $x_3 = \mu$ result from equilibria and mass balances:

$$2x_1 + x_2 + \frac{BP\,x_1}{x_2\,fa} + \frac{BR\,x_1}{x_2\,fa^2} + \frac{2QQ\,x_1}{fa} + 3K_3\,x_1\,x_2 + 5N\,x_1^2\,x_2 + \frac{6P(x_1\,x_2\,fa)^2}{ft} + \frac{2QJ\,x_1}{ft} - T = 0 \quad \text{(Eq. 16)}$$

$$x_1^2\left(N\,x_2^2 + 2P\,x_2^3\right) + x_1[K_3\,x_2^2 - BP - BR] + x_2\,D - x_2^2 = 0 \quad \text{(Eq. 17)}$$

$$x_2 + K_3\,x_1\,x_2 + BR\frac{x_1}{x_2} + 2\,N\,x_1^2\,x_2 + 3P\,x_1^2\,x_2^2 + 3QJ\,x_1 - x_3 = 0 \quad \text{(Eq. 18)}$$

Eqs. 16 and 18 were derived from the total iodine atoms balance and the ionic strength, respectively. **Eq. 17** follows from:

$$\begin{cases} x_2(C - x_1) = x_1[K_3\,x_2^2 + BP + BR + x_2(QQ + QJ)] + 2x_1^2\,x_2^2(N + P\,x_2) \\ x_2(C - x_1) = x_1[2BP + 2BR + x_2(QQ + QJ)] + 2N\,x_1^2\,x_2^2 + D\,x_2 - x_2^2 \end{cases} \quad \text{(Eq. 19)}$$

The five unknowns [I_2], [I^-], μ, *fa* and *ft* were computed at each of the given pH values solving a system including **Eqs. 16-18** and the two formulas that return the activity coefficients from **Eqs. 14-15**. All these equations were set to equal zero and the solutions were computed iteratively between bounds $0 \leq x_1 \leq C$, $0 \leq x_2 \leq T$, $0 \leq x_3 \leq T + 7C$, $0 \leq fa \leq 2$, $0 \leq ft \leq 2$. All of the other concentrations were computed according to the previously listed equilibria.

The absolute value of the residual, |Δ|, was calculated as the difference between the sum of iodine atoms in all of the computed iodine species and *T* (**Table S1**).

Appendix 2 – MATLAB code for iodine speciation

```
New/function
function F = f1(x)
global P N K3 BP BR D QQ QJ T
F(1) = (x(1).^2)*(2*P*((x(4).^2)./x(5))*(x(2).^3)+N*(x(2).^2))+x(1)*(K3*(x(2).^2)-
(BP./x(4))-(BR./(x(4).^2)))+(x(2).^2)-x(2)*D;
F(2) =
2*x(1)+x(2)+BP*x(1)*(1./x(4))*(1./x(2))+BR*x(1)*(1./(x(4).^2))*(1./x(2))+2*QQ*x(1)*
(1./x(4))+3*K3*x(1)*x(2)+5*N*(x(1).^2)*x(2)+6*P*((x(4)*x(1)*x(2)).^2)*(1./x(5))+2*Q
J*x(1)*(1./x(5))-T;
F(3) =
x(2)+K3*x(1)*x(2)+((BR./(x(4).^2))*x(1)./(x(2)))+N*(x(1).^2)*x(2)+3*P*((x(4).^2)./x
(5))*(x(1).^2)*(x(2).^2)+ 3*(QJ./x(5))*x(1)-x(3);
F(4) = log(x(4))+0.3.*x(3)+0.509*(x(3).^2)./(1 + 0.328*3*(x(3).^0.5));
F(5) = log(x(5))+0.3.*x(3)+0.509*4*(x(3).^2)./(1 + 0.328*4*(x(3).^0.5));
end
save as f1.m

New/function
function [c,ceq] = fminconstr(x)

c = []; %no nonlinear inequality
ceq = f1(x); %the fsolve objective is fmincon constraints
save as fminconstr.m

Workspace
%Constants
K1 =5.44*1e-13;
K2 =2.3*1e-11;
K3 =723;
K5 =9;
K6 =0.1;
K7 =0.13;
K8 =3.3*1e-15;
N = K3*K5;
P =(K3^2)*K6;

%Options
options = optimoptions(@fmincon,'Display','iter','Algorithm','sqp',
'Hessian','bfgs','MaxFunEvals',100000,'MaxIter',5000,'TolPCG',1e-12,'TolFun',1e-
12,'TolX',1e-12);

%Variables
C=0.05; % added iodine
D=0.5; % added iodide
T=2*C+D; % total iodine atoms
H=10^(-7); % proton concentration - run one computation per pH point
BP= K1./H;
BR=BP*K2./H;
QQ= K1*K2*K7*1e14./H;
QJ= QQ*K8./H;

%Bounds [free iodine; free iodide; ionic strength; fa; ft]
lb=[0;0;0;0;0];
ub=[C;T;D+ 7*C;2;2];

%Initial guess [free iodine; free iodide; ionic strength; fa; ft]
x0=[C;D;D;1;1];

global P N K3 BP BR D QQ QJ T
% Call solver
[x,fval] = fmincon(@(x)0,x0,[],[],[],[],lb,ub,@fminconstr,options)
```

```
%Once you've got x, compute:

%Equilibrium concentrations
A(1)=x(1); % free iodine
A(2)=x(2); % free iodide

A(3)=K1*A(1)./(H*A(2)*x(4)); % HIO
A(4)=K2*A(3)./(H*x(4)); % IO-
A(5)=K7*1e14*H* A(2)*A(4)*x(4); % HI2O-
A(6)=K3*A(1)*A(2); % I3-
A(7)=K5*A(6)*A(1); % I5-
A(8)=K6*(A(6).^2)*(x(4).^2)./x(5); % I62-
A(9)=K8*A(5)*x(4)./(H*x(5)); % I2O2-

A(10)=2*A(1)+A(2)+A(3)+A(4)+2*A(5)+3*A(6)+5*A(7)+6*A(8)+2*A(9)- T; % residuals
```

Appendix 3 – MATLAB code for dummy cells

```
New/function
function ZcircuitoPtPt=ZcircuitoPtPt(A,Freq)
%This function returns the modelled complex impedance of a dummy cell..
%Pt|electrolyte|Pt. Feed it with: Freq=array of frequencies in Hz;..
%A=array of ordered parameters=[Rs;Rpt;Rd;D/L^2;Tpt;fipt] in..
%[Ohm;Ohm;Ohm;s^fipt/Ohm;s^-1;dimensionless]
Zd = A(3)*tanh(((1/A(4))*1i*Freq*2*pi).^0.5)./(((1/A(4))*1i*Freq*2*pi).^0.5);
RZCPE = A(5)*((1i*Freq*2*pi).^A(6));
ZcircuitoPtPt = A(1)+(1./((1./(A(2)+Zd))+(RZCPE)));
end
save as Zcircuito.m

New/function
function leastsqrs=leastsqrs(A,Freq,Zre,Zim)
%This function allows to run the fit minimizing difference between the modelled..
%circuit impedance Zmod and the experimental values of Zexp as a function of Freq..
%The problem is solved in terms of weighted least squares, that is, the function
to..
%minimize is the modulus of sum(weight*(Zmod-Zexp).^2). The weight matrix is..
%1/Zexp.Feed this with function with: Zim and Zre = arrays of the experimental..
%impedance components in Ohm; A0 = initial guess for the parameters. A0 must be
an..
%array of 6 real numbers corresponding to the goal parameters..
%[Rs;Rpt;Rd;D/L^2;Tpt;fipt];[lb] and [ub]= arrays of lower and upper bounds of
the..
%same size as A0. Bounds are referred to the respective ordered parameters in A0).
Z=Zre+(1i*Zim);
W=1./Z;
Zcircuito = ZcircuitoPtPt(A,Freq);
leastsqrs=sum(abs(W.*((Zcircuito-Z).^2)));
end
save as leastsqrs.m

New/function
function [Output,Results] = outputdata (Aes,fval,Freq,Zre,Zim,L,Sample)
%Run this after calling leastsqrs(A,Freq,Zre,Zim). Feed this with: L=1/2 Surlyn..
%thickness in cm; Sample={samplename}
RptSE=Aes(2)/2; %Ohm, referred to one single electrode
CdlDUMMY=(Aes(5)/(Aes(2))^(Aes(6)-1))^(1/Aes(6)); %Farad
CdlSE=CdlDUMMY*2; %Farad, referred to one single electrode
D=Aes(4)*(L^2);%diffusion coefficient of triiodide in cm^2/s
Rs=Aes(1);%Ohm
Rpt=Aes(2);%Ohm
Rd=Aes(3); %Ohm
Tpt=Aes(5);%s^fipt/Ohm
fipt=Aes(6);
ModelledBodePhase=rad2deg(angle(ZcircuitoPtPt(Aes,Freq)));
ModelledZre=real(ZcircuitoPtPt(Aes,Freq));
ModelledZim=imag(ZcircuitoPtPt(Aes,Freq));
ExpBodePhase=rad2deg(angle(Zre+(1i*Zim)));
Output=table(Freq,ExpBodePhase,Zre,Zim,ModelledBodePhase,ModelledZre,ModelledZim);
Results=table(Rs,Rpt,Rd,Tpt,fipt,RptSE,CdlDUMMY,CdlSE,D,fval,'Rowname',Sample);
figure %Bode plot, modelvsExp
semilogx(Freq,-1*ModelledBodePhase,Freq,-1*ExpBodePhase,'g*')
figure %Nyquist plot, modelvsExp
plot(ModelledZre,-1*ModelledZim,Zre,-1*Zim,'g*')
end
save as outputdata.m

Workspace
%Options
```

```
options = optimoptions(@fmincon,'Display','iter','Algorithm','sqp',
'Hessian','bfgs','MaxFunEvals',100000,'MaxIter',5000,'TolPCG',1e-12,'TolFun',1e-
12,'TolX',1e-12);

%call finconstr
[Aes,fval,exitflag]=fmincon(@(A)leastsqrs(A,Freq,Zre,Zim),A0,[],[],[],[],lb,ub,@(A)
stopPtPt(A,Freq,Zre,Zim),options);

%Run the following to display the output tables
[Output,Results] = outputdata (Aes,fval,Freq,Zre,Zim,L,Sample)
```

Appendix 4 - MATLAB code for simplified equivalent circuit without mass transport (for cells containing EL-HSE)

```
New/function
function ZcircuitoHSE=ZcircuitoHSE(A,Freq)
%This function returns the modelled complex impedance of a cell modelled as..
%Rs(CPEpt(//)Rpt)(CPEtio2(//)Rct) where (//) stands for a parallel connection..
%Feed it with: Freq=array of frequencies in Hz;..
%A=array of ordered parameters=[Rs;Rpt;TTiO2;fiTiO2;Tpt;fipt;Rct] in..
%[Ohm;Ohm;s^fiTiO2/Ohm;dimensionless;s^fipt/Ohm;dimensionless;Ohm]
RZCPETiO2 = A(3)*((1i*Freq*2*pi).^A(4));
RZCPEpt = A(5)*((1i*Freq*2*pi).^A(6));
ZcircuitoHSE = A(1)+(1./((1./A(2))+(RZCPEpt)))+(1./((1./A(7))+(RZCPETiO2)));
end
save as ZcircuitoHSE.m

New/function
function leastsqrs=leastsqrsHSE(A,Freq,Zre,Zim)
%This function allows to run the fit minimizing difference between the modelled..
%circuit impedance Zmod and the experimental values of Zexp as a function of Freq..
%The problem is solved in terms of weighted least squares, that is, the function
to..
%minimize is the modulus of sum(weight*(Zmod-Zexp).^2). The weight matrix is..
%1/Zexp.Feed this with function with: Zim and Zre = arrays of the experimental..
%impedance components in Ohm; A0 = initial guess for the parameters. A0 must be
an..
%array of 7 real numbers corresponding to the goal parameters..
%[Rs;Rpt;TTiO2;fiTiO2;Tpt;fipt;Rct];[lb] and [ub]=arrays of lower and upper
bounds..
%of the same size as A0.Bounds are referred to the respective ordered parameters in
A0
Z=Zre+(1i*Zim);
W=1./Z;
Zcircuito = ZcircuitoHSE(A,Freq);
leastsqrsHSE=sum(abs(W.*((Zcircuito-Z).^2)));
end
save as leastsqrsHSE.m

New/function
function [Output,Results] = outputdataHSE (Aes,fval,Freq,Zre,Zim,Sample)
%Run this after calling leastsqrs(A,Freq,Zre,Zim). Feed it with Sample={samplename}
Rs=Aes(1);%Ohm
Rpt=Aes(2);%Ohm
TTiO2=Aes(3);%s^fiTiO2/Ohm
fiTiO2=Aes(4);
Tpt=Aes(5);%s^fipt/Ohm
fipt=Aes(6);
Rct=Aes(7); %Ohm
Cdlpt=(Aes(5)/(Aes(2))^(Aes(6)-1))^(1/Aes(6));%Farad
CdlTiO2=(Aes(3)/(Aes(7))^(Aes(4)-1))^(1/Aes(4));%Farad
tauele=Rct*CdlTiO2;%effective electron lifetime in s
ModelledBodePhase=rad2deg(angle(ZcircuitoHSE(Aes,Freq)));
ModelledZre=real(ZcircuitoHSE(Aes,Freq));
ModelledZim=imag(ZcircuitoHSE(Aes,Freq));
ExpBodePhase=rad2deg(angle(Zre+(1i*Zim)));
Output=table(Freq,ExpBodePhase,Zre,Zim,ModelledBodePhase,ModelledZre,ModelledZim);
Results=table(Rs,Rpt,TTiO2,fiTiO2,Tpt,fipt,Rct,Cdlpt,CdlTiO2,tauele,fval,'Rowname',
Sample)
figure % Bode plot, modelvsExp
semilogx(Freq,-1*ModelledBodePhase,Freq,-1*ExpBodePhase,'g*')
figure %Nyquist plot, modelvsExp
plot(ModelledZre,-1*ModelledZim,Zre,-1*Zim,'g*')
end
```

save as outputdataHSE.m

Workspace

```
% Options
options = optimoptions(@fmincon,'Display','iter','Algorithm','sqp',
'Hessian','bfgs','MaxFunEvals',10000,'MaxIter',5000,'TolPCG',1e-15,'TolFun',1e-
15,'TolX',1e-15);

% call finconstr
[Aes,fval,exitflag]=fmincon(@(A)leastsqrsHSE(A,Freq,Zre,Zim),A0,[],[],[],[],lb,ub,[
],options);

%Run the following to display the output tables
[Output,Results] = outputdataHSE (Aes,fval,Freq,Zre,Zim,Sample)
```

Appendix 5 – MATLAB code for simplified equivalent circuit

```
New/function
function ZcircuitoSIMP=ZcircuitoSIMP(A,Freq)
%This function returns the complex impedance of a cell modelled as..
%Rs(CPEpt(//)Rpt Zd)(CPEtio2(//)Rct) where (//) stands for a parallel connection..
%Feed it with: Freq=array of frequencies in Hz;..
%A=array of ordered parameters=[Rs;Rpt;TTiO2;fiTiO2;Cdl;Rct;D/L^2;Rd] in..
%[Ohm;Ohm;s^fiTiO2/Ohm;dimensionless;F;Ohm;s^-1;Ohm]
Zd =A(8)*tanh(((1/A(7))*1i*Freq*2*pi).^0.5)./(((1/A(7))*1i*Freq*2*pi).^0.5);
RZCdl =A(5)*1i*Freq*2*pi;
RZCPETiO2= A(3)*((1i*Freq*2*pi).^A(4));
ZcircuitoSIMP =A(1)+(1./((1./(A(2)+Zd))+RZCdl))+(1./((1./A(6))+RZCPETiO2));
end
save as ZcircuitoSIMP.m

New/function
function leastsqrs=leastsqrsSIMP(A,Freq,Zre,Zim)
%This function allows to run the fit minimizing difference between the modelled..
%circuit impedance Zmod and the experimental values of Zexp as a function of Freq..
%The problem is solved in terms of weighted least squares, that is, the function
to..
%minimize is the modulus of sum(weight*(Zmod-Zexp).^2). The weight matrix is..
%1/Zexp.Feed this with function with: Zim and Zre = arrays of the experimental..
%impedance components in Ohm; A0 = initial guess for the parameters. A0 must be
an..
%array of 8 real numbers corresponding to the goal parameters..
%[Rs;Rpt;TTiO2;fiTiO2;Cdl;Rct;D/L^2;Rd];[lb] and [ub]=arrays of lower and upper..
%bounds of the same size as A0.Bounds are referred to the respective ordered..
%parameters in A0
Z=Zre+(1i*Zim);
W=1./Z;
Zcircuito = ZcircuitoSIMP(A,Freq);
leastsqrsSIMP=sum(abs(W.*((Zcircuito-Z).^2)));
end
save as leastsqrsSIMP.m

New/function
function [Output,Results] = outputdataSIMP (Aes,fval,Freq,Zre,Zim,L,Sample)
%Run this after calling leastsqrs(A,Freq,Zre,Zim). Feed this with: L=1/2 Surlyn..
%thickness in cm; Sample={samplename}
Rs=Aes(1);%Ohm
Rpt=Aes(2);%Ohm
TTiO2=Aes(3);%s^fiTiO2
fiTiO2=Aes(4);
Cdl=Aes(5);%Farad
Rct=Aes(6);%Ohm
Rd=Aes(8);%Ohm
CdlTiO2=(Aes(3)/(Aes(6))^(Aes(4)-1))^(1/Aes(4)); %Farad
D=Aes(7)*(L^2);% diffusion coefficient of triiodide in cm^2/s
tauele=Rct*CdlTiO2;%effective electron lifetime in s
ModelledBodePhase=rad2deg(angle(ZcircuitoSIMP(Aes,Freq)));
ModelledZre=real(ZcircuitoSIMP(Aes,Freq));
ModelledZim=imag(ZcircuitoSIMP(Aes,Freq));
ExpBodePhase=rad2deg(angle(Zre+(1i*Zim)));
Output=table(Freq,ExpBodePhase,Zre,Zim,ModelledBodePhase,ModelledZre,ModelledZim);
Results=table(Rs,Rpt,TTiO2,fiTiO2,Cdl,Rct,Rd,D,tauele,CdlTiO2,fval,'Rowname',Sample
)
figure % Bode plot, modelvsExp
semilogx(Freq,-1*ModelledBodePhase,Freq,-1*ExpBodePhase,'g*')
figure %Nyquist plot, modelvsExp
plot(ModelledZre,-1*ModelledZim,Zre,-1*Zim,'g*')
end
save as outputdataSIMP.m
```

```
Workspace
% Options
options = optimoptions(@fmincon,'Display','iter','Algorithm','sqp',
'Hessian','bfgs','MaxFunEvals',100000,'MaxIter',5000,'TolPCG',1e-15,'TolFun',1e-
15,'TolX',1e-15);

% call finconstr
[Aes,fval,exitflag]=fmincon(@(A)leastsqrsSIMP(A,Freq,Zre,Zim),A0,[],[],[],[],lb,ub,
[],options);

%Run the following to display the output tables
[Output,Results] = outputdataSIMP (Aes,fval,Freq,Zre,Zim,L,Sample)
```

References

[1] W. Gottardi, *Iodine and Iodine compounds*, in S. Block, *Disinfection sterilization and preservation*, Lippincott Williams & Wilkins, Philadelphia, 2001, 159-184.

[2] A. Stearn, *J. Am. Chem. Soc.*, 1922, **44**, 670-678.

[3] J. Chambers, J. Stokes and R. Stokes, *J. Phys. Chem.*, 1956, **60**, 985-986.

[4] G. Boschloo and A. Hagfeldt. *Acc. Chem. Res.*, 2009, **42**, 1819-1826.

[5] W. Gottardi, *Arch. Pharm.*, 1999, **332**, 151-157.

[6] R. Ramette and R. Sandford, *J. Am. Chem. Soc.*, 1965, **87**, 5001-5005.

[7] D. Palmer, R. Ramette and R. Mesmer, *J. Solution Chem.*, 1984, **13**, 673-683.

[8] K. H. Taik and W. J. Frederick Jr., *J. Chem. Eng. Data*, 1988, **33**, 177-184.

[9] A. Casas, S. Omar, J. Palomar, M. Oliet, M. V. Alonso and F. Rodriguez, *RSC Adv.*, 2013, **3**, 3453-3460.

[10] W. Gottardi, *Fresenius' J. Anal. Chem.*, 1998, **362**, 263-269.
